# Comparative Microsatellite Typing of New World *Leishmania infantum* Reveals Low Heterogeneity among Populations and Its Recent Old World Origin

**DOI:** 10.1371/journal.pntd.0001155

**Published:** 2011-06-07

**Authors:** Katrin Kuhls, Mohammad Zahangir Alam, Elisa Cupolillo, Gabriel Eduardo M. Ferreira, Isabel L. Mauricio, Rolando Oddone, M. Dora Feliciangeli, Thierry Wirth, Michael A. Miles, Gabriele Schönian

**Affiliations:** 1 Institut für Mikrobiologie und Hygiene, Charité Universitätsmedizin Berlin, Berlin, Germany; 2 Laboratório de Pesquisa em Leishmaniose, Instituto Oswaldo Cruz - Fiocruz, Rio de Janeiro, Brazil; 3 Laboratório de Biologia Molecular de Insetos, Instituto Oswaldo Cruz - Fiocruz, Rio de Janeiro, Brazil; 4 Department of Pathogen Molecular Biology, Faculty of Infectious and Tropical Diseases, London School of Hygiene and Tropical Medicine, London, United Kingdom; 5 Instituto de Investigaciones en Ciencias de la Salud, Universidad Nacional de Asuncion, Asuncion, Paraguay; 6 Universidad de Carabobo, BIOMED-Centro Nacional de Referencia de Flebotomos de Venezuela, UC-BIOMED-CNRFV, Maracay, Venezuela; 7 Département de Systématique et Évolution, Ecole Pratique des Hautes Etudes, Muséum National d'Histoire Naturelle, UMR-CNRS 5202, Paris, France; National Institutes of Health, United States of America

## Abstract

*Leishmania infantum* (syn. *L. chagasi*) is the causative agent of visceral leishmaniasis (VL) in the New World (NW) with endemic regions extending from southern USA to northern Argentina. The two hypotheses about the origin of VL in the NW suggest (1) recent importation of *L. infantum* from the Old World (OW), or (2) an indigenous origin and a distinct taxonomic rank for the NW parasite. Multilocus microsatellite typing was applied in a survey of 98 *L. infantum* isolates from different NW foci. The microsatellite profiles obtained were compared to those of 308 *L. infantum* and 20 *L. donovani* strains from OW countries previously assigned to well-defined populations. Two main populations were identified for both NW and OW *L. infantum*. Most of the NW strains belonged to population 1, which corresponded to the OW MON-1 population. However, the NW population was much more homogeneous. A second, more heterogeneous, population comprised most Caribbean strains and corresponded to the OW non-MON-1 population. All Brazilian *L. infantum* strains belonged to population 1, although they represented 61% of the sample and originated from 9 states. Population analysis including the OW *L. infantum* populations indicated that the NW strains were more similar to MON-1 and non-MON-1 sub-populations of *L. infantum* from southwest Europe, than to any other OW sub-population. Moreover, similarity between NW and Southwest European *L. infantum* was higher than between OW *L. infantum* from distinct parts of the Mediterranean region, Middle East and Central Asia. No correlation was found between NW *L. infantum* genotypes and clinical picture or host background. This study represents the first continent-wide analysis of NW *L. infantum* population structure. It confirmed that the agent of VL in the NW is *L. infantum* and that the parasite has been recently imported multiple times to the NW from southwest Europe.

## Author Summary

Leishmaniasis is a vector borne disease with a broad spectrum of clinical forms caused by protozoan parasites of the genus *Leishmania*. Visceral leishmaniasis is the most severe, systemic form of the disease. It is caused by parasites belonging to the *Leishmania donovani* complex, which includes *L. infantum* and *L. donovani* in the Old World (OW) and *L. infantum* (syn. *L. chagasi*) in the New World (NW). The identity and origin of the causative agent of VL in the Americas have been the subjects of much debate for decades. Different scientific approaches led to different conclusions, either favouring the hypothesis of indigenous origin of this parasite and its status as distinct species, or a recent importation of *L. infantum* by European colonists and synonymy of *L. infantum* and *L. chagasi*. We performed the first broad population study of these parasites from the NW using highly variable microsatellite markers. The level of heterogeneity and population structure was very low in contrast to the OW. Using a combined data analysis of NW and OW strains we have provided conclusive evidence of recent multiple introductions of *L. infantum* from Southwest Europe into the New World and for synonymy of *L. infantum* and *L. chagasi*.

## Introduction

In 1937 the causative agent of visceral leishmaniasis (VL) in the New World (also referred to as American visceral leishmaniasis – AVL) was designated as a distinct species, *Leishmania (L.) chagasi* Cunha & Chagas [1]. Many studies have subsequently concluded that the causative agent is indistinguishable from *L. infantum*, derived from Europe [2]–[4]. To explore the molecular epidemiology of AVL, we have applied a high resolution population genetic analysis to a vast collection of New World (NW) and Old World (OW) isolates.

Visceral leishmaniasis in the New World extends from the southern parts of the USA [5], [6] and Mexico to the North of Argentina, including countries such as Brazil, Paraguay, Bolivia, Venezuela, Suriname, Guyana, Colombia, Honduras, Panama, Costa Rica, El Salvador, Guadeloupe, Guatemala, and Nicaragua [7]–[11]. Brazil is the country that accounts for the highest number (∼90%) of cases [12], [13]. The principal foci are located in drier, poorly forested areas, although there are several foci in the densely forested Amazon region and the Guianan Ecoregion Complex (GEC), which covers some States of Venezuela and all of Guyana, Suriname and French Guiana and the upper parts of the Brazilian states Amazonas, Roraima, Pará, and Amapá. The main foci here are in Pará, Roraima (Brazil), Bolivar (Venezuela) and parts of Guyana. There are few cases reported from Suriname and no cases from French Guiana except a recently imported canine case [14]–[17].

To a lesser extent, NW *L. infantum* also causes atypical cutaneous leishmaniasis (atypical CL). This clinical manifestation has been reported since the 1970s mainly from Caribbean countries such as Venezuela, Honduras, Costa Rica, Nicaragua, but sporadically also from Brazil [18]–[23]. Except in Brazil, atypical CL cases are characterised by non-ulcerative skin lesions that are often misidentified as nodular infantile tuberculoid leprosy. Host immuno-genetic factors and/or parasite factors in combination with socio-economical and environmental factors are likely to play a role in determining the varied clinical picture, as in the case of Mediterranean *L. infantum* infections.

In the NW, domestic dogs are primary reservoirs of infection for humans, but foxes (*Cerdocyon thous*), native marsupials (*Didelphis marsupialis*, *D. albiventris*) and rodents (e.g. domestic rats) have also been found infected not only in urban areas but also in the Amazonian region [11], [14], [15]. The sand fly *Lutzomyia longipalpis* is the primary vector of *L. infantum* in the NW [12], [15], however, differences in the sand fly populations [24]–[28] and perhaps also the involvement of other sand fly species (e.g. *Lu. evansi*) [11],[15],[29],[30] may contribute to the variable clinical manifestations of the disease seen in different geographic regions.

Taxonomically NW *L. infantum* (syn. *L. chagasi*) belongs to the *L. donovani* species complex of the subgenus *Leishmania* Ross 1903, which in addition includes *L. infantum* and *L. donovani* from the OW [7]. There are two different hypotheses on the origin of NW *L. infantum*, of which the first one is now widely accepted: (1) *L. infantum* has been imported from Europe during the Spanish and Portuguese colonization carried by dogs or rats, and (2) *L. chagasi* is indigenous to the Americas [8], [31]–[36]. As summarized by Dantas-Torres [34], [35] these two hypotheses have led to much confusion regarding the nomenclature and at least 6 different nomenclatures are used in the literature. Until now, there have been no extensive studies of the population structure of NW *L. infantum* with a reasonable number of strains from different regions, environments, hosts and reservoirs and, therefore the taxonomic status of NW *L. infantum* is still not clear.

To detect structure of *Leishmania* populations it is essential to use a typing method with a high discriminatory potential. Many previously used methods were not adequate for discriminating at this taxonomic level. One of the most powerful and discriminative DNA-based methods for strain differentiation and population genetics is the analysis of highly variable, co-dominant microsatellite markers. Recently, multilocus microsatellite typing (MLMT) has been used successfully to differentiate *L. infantum* populations in the Mediterranean region of Europe and North Africa, the Middle East and Uzbekistan [4], [37]–[41] as well as *L. donovani* populations in the Indian subcontinent and East Africa [42], [43]. This method enabled differentiation even at the intra-zymodeme level, as shown for the predominant MON-1 zymodeme of *L. infantum*.

In the present study we have applied MLMT for an extensive population survey of NW *L. infantum* originating mainly from different endemic regions within Brazil, but also from other countries. To our knowledge this is the first comprehensive study of population structure of *L. infantum* in the NW. We show that NW *L. infantum*, indeed, was introduced on multiple occasions in recent times from European source populations of *L. infantum* and is inseparable from them. We also provide substantial new insight into the molecular epidemiology of AVL.

## Materials and Methods

### Parasite cultures and DNA extraction

**Figure 1:**
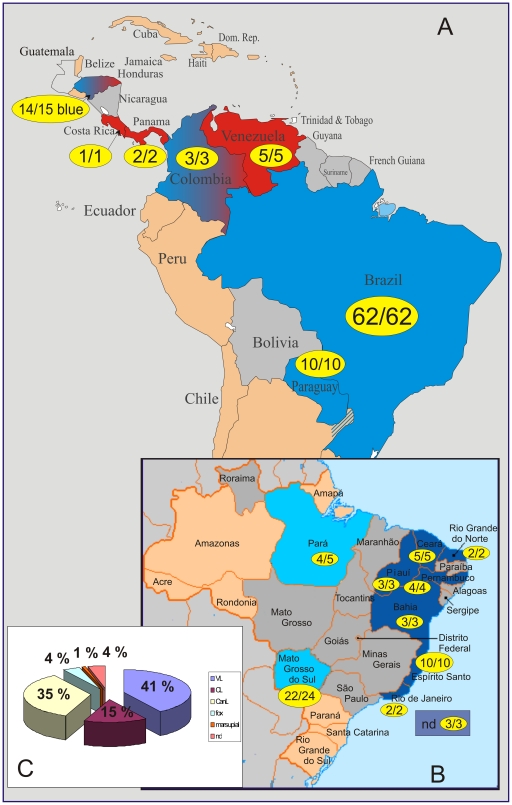
Distribution of the populations and subpopulations of NW *L. infantum* in the studied endemic regions. The first number refers to the number of strains belonging to the indicated population (specified by the color), the second number gives the overall number of studied strains from the respective region. Additional endemic regions that were not studied are marked by grey color. (**A**): Colors of the respective population correlate with those in Fig. 2 (Pop1-INF_NW_ - blue; Pop2-INF_NW_ - red). In Honduras all but one strain belong to Population 1. In Colombia shared memberships in both populations could be recognized, with the predominant part of membership in Population 1. (**B**): Sub-populations found in Population 1. Colors correlate with those of the sub-populations in Fig. 2 (Sub-Pop1A-INF_NW_ - dark blue, Sub-Pop1B-INF_NW_ - light blue). In Pará one strain belongs to Sub-Pop1A-INF_NW_, and two strains have shared membership in both sub-populations 1A and 1B with predominating 1B traits, and two strains belong to sub-population 1B. Strains from Colombia belong to the 1B subpopulation (light blue), the Hondurian strains to 1A (dark blue) (not shown). (**C**): Distribution of clinical pictures among the 98 studied strains. VL – visceral leishmaniasis, CL – cutaneous leishmaniasis, CanL – canine leishmaniasis, nd – not determined.

Sources, designation, geographical origins, MLEE identification, if known, and clinical manifestation for the 426 studied strains, including NW *L. infantum*, OW *L. infantum* and *L. donovani* are listed in **Table S1**. NW *L. infantum* was represented by 98 strains from Brazil, Paraguay, Colombia, Venezuela, Honduras, Panama, and Costa Rica (**Figure 1A**, **Table 1**). Most NW *L. infantum* came from Brazil and **Figure 1B** shows the number of strains used from the respective Brazilian endemic regions. **Figure 1C** depicts the percentages of NW *L. infantum* strains causing different clinical pictures. The 308 *L. infantum* from seven European and two North African Mediterranean countries, four countries from the Middle East and Asia, as well as 20 *L. donovani* strains from East Africa and India were analysed in previous population genetic studies and have been incorporated here to elucidate the phylogenetic position of NW *L. infantum* in relation to OW *L. donovani* complex species. **Table 1** summarises the number of strains per species according to geographical origin (continent and country), zymodeme and clinical picture. Most of the Brazilian strains were obtained from the *Leishmania* collection of the Oswaldo Cruz Institute (CLIOC, WDCM731, http://clioc.ioc.fiocruz.br). All strains from CLIOC were typed by MLEE as IOC/Z1 which corresponds to zymodeme MON-1 [44] (unpublished data) (**Table S1**). Strains from Paraguay were collected in 2000 (Programa Nacional de Leishmaniosis, SENEPA, Ministry of Public Health, Paraguay), and the strains from Venezuela were provided by the Universidad de Carabobo, Centro Nacional de Referencia de Flebotomos de Venezuela (CNRFV-BIOMED-UC). DNA of strains from Panama, Costa Rica and some from Brazil were obtained from the Royal Tropical Institute (KIT), Amsterdam, The Netherlands and from the WHO's Jerusalem Reference Centre for Leishmaniases, Hebrew University – Hadassah Medical School, Jerusalem. Strains from Honduras, which were previously typed by kDNA-RFLP [20] were given by the London School of Hygiene and Tropical Medicine, London, UK.

**Table 1:** Numbers of strains per species, region, clinical picture and host of the studied sample set.

species	continent	country	Strains overall	VL	VL/ HIV+	CL	CanL	fox	*Didel-phis*	Sand fly	nd	zymodeme
*L. infantum*	Central	Costa Rica	**1**								**1**	nd
NW	America	Panama	**2**			**2**						nd
(total 98)		Honduras	**15**	**2**		**13**						nd
	South America	Venezuela	**5**	**3**		**1**	**1**					nd
		Colombia	**3**	**1**			**1**		**1**			nd
		Paraguay	**10**	**2**			**7**				**1**	nd
		Brazil	**62**	**28**	**3**		**25**	**4**			**2**	MON-1, IOC/Z1, nd
		Rio de Janeiro	3		2		1					
		Espírito Santo	10	5			5					
		Ceará	5	2			3					
		Bahia	3	1			2				2	
		Pará	5	1				4				
		Rio Grande do Norte	2				2					
		Pernambuco	4	1			3					
		Piauí	3	1			2					
		Mato Grosso do Sul	24	16	1		7					
		nd	3	1								
*L. Infantum* OW	Europe	Spain	**65**	**6**	**27**	**2**	**30**					MON-1,24,27,34, 37,183,198, 199
(total 308)		Portugal	**44**	**6**	**12**	**2**	**20**	**1**		**3**		MON-1, 80
		France	**32**	**21**	**2**	**7**	**3**					MON-1, 11, 29, 108
		Italy	**30**	**8**	**7**	**2**	**9**	**1 cat**		**3**		MON-1,188, 228
		Greece	**15**	**9**	**1**		**5**					MON-1, 98
		Turkey	**2**				**1**				**1**	MON-1
		Malta	**1**			**1**						MON-78
	Africa	Tunisia	**24**	**16**		**8**						MON-1, 24
		Algeria	**41**	**18**		**11**	**12**					MON-1, 24, 80
	Middle East	Israel	**27**	**1**			**26**					MON-1
		Palestine	**10**	**10**								MON-1, 281
	Asia	Uzbekistan	**14**	**6**			**8**					MON-1
		China	**3**	**2**							**1**	nd
*L. donovani*	Africa	Sudan	**8**	**7**							**1**	MON-30,82,257,258, 274
(total 20)		Ethiopia	**2**	**2**								MON-18, 31
		Kenya	**5**	**1**		**2 PKDL**					**2**	MON-37, nd
	Asia	India	**5**	**4**							**1**	MON-2, nd

VL – visceral leishmaniasis, CL – cutaneous leishmaniasis, PKDL – post Kala Azar dermal leishmaniasis, CanL – canine leishmaniasis, nd – not defined, MON – zymodeme according to the Montpellier MLEE typing system [86], IOC/Z –zymodemes according to the CLIOC system [44], with zymodeme IOC/Z1 corresponding to MON-1.

DNA was isolated using proteinase K- phenol/chloroform extraction [45] or the Wizard™ Genomic DNA Purification System (Promega, Madison, WI, USA) according the manufacturer's protocol, suspended in TE-buffer or distilled water and stored at 4°C until use.

### PCR amplification assays and electrophoretic analysis of the microsatellite markers

The standard set of 14 primer pairs (Lm2TG, TubCA, Lm4TA, Li41-56, Li46-67, Li22-35, Li23-41, Li45-24, Li71-33, Li71-5/2, Li71-7, CS20, kLIST7031, LIST7039) that we have previously applied for the *L. donovani* complex was used for amplification of microsatellite containing fragments, as previously described [38], [43]. PCRs were performed with fluorescence-conjugated forward primers. Screening of length variations of the amplified markers was done by automated fragment analysis using capillary sequencers. PCR products from amplified microsatellites were analysed either with the fragment analysis tool of the CEQ 8000 automated genetic analysis system (Beckman Coulter, USA) or the ABI PRISM GeneMapper (Applied Biosystems, Foster City, CA).

### Data analysis

Population structure was investigated by the STRUCTURE software [46], which applies a Bayesian model-based clustering approach. This algorithm identifies genetically distinct populations on the basis of allele frequencies. Genetic clusters are constructed from the genotypes identified, estimating for each strain the fraction of its genotype that belongs to each cluster. This clustering method proved superior to distance-based approaches for processing data sets of low variability like those presented by *L. infantum*. The following parameters were used: “burn-in” period of 20,000 iterations, probability estimates based on 200,000 Markov Chain Monte Carlo iterations. The most appropriate number of populations was determined by calculation of Δ*K*, which is based on the rate of change in the log probability of data between successive *K* values [47].

Phylogenetic analysis was based on microsatellite genetic distances, calculated with the program POPULATIONS 1.2.28 (http://bioinformatics.org/~tryphon/populations) for the numbers of repeats within each locus using the Chord-distance [48], which follows the infinite allele model (IAM). Neighbor-joining trees were constructed with the POPULATIONS software and visualized with MEGA [49]. Microsatellite markers as well as populations were analysed with respect to diversity of alleles (A), expected (gene diversity) and observed heterozygosity (*H*_e_ and *H*_o_, respectively), and the inbreeding coefficient *F*_IS_ applying GDA (http://hydrodictyon.eeb.uconn.edu/people/plewis/software.php).

Genetic differentiation and gene flow was assessed by *F*-statistics [50] calculating the *F*_ST_ (theta) values (IAM) [51] with the corresponding p-values (confidence test) using the MSA software [52] (for details see **Methods S1**).

### Ethics statement

Research in this study was subject to ethical review by the European Commission and approved as part of contract negotiation for Project LeishEpiNetSA (contract 01547): the work conformed to all relevant European regulations. The research was also reviewed and approved by the ethics committee of the London School of Hygiene and Tropical Medicine (approval 5092). The *Leishmania* strains analysed in this consolidated microsatellite analysis were principally reference strains derived from international cryobanks as CLIOC (registered at the World Data Centre for Microorganisms under the number WDCM731 and recognized as depository authority by the Brazilian Ministry of the Environment, MMA/CGEN Deliberação CGEN 97 de 22/03/2005, Processo 02000.003672/2004-34), the cryobank of the London School of Hygiene and Tropical Medicine (LSHTM), the Centro Nacional de Referencia de Flebotomos de Venezuela (CNRFV-BIOMED-UC), the cryobank of the Royal Tropical Institute (KIT) in Amsterdam, Netherlands and the WHO's Jerusalem Reference Centre for Leishmaniases, Hebrew University, Jerusalem, Israel. They have already been object of many publications. Several other strains were from small prior studies also using other methods, such as the strains from Honduras that were isolated 16–23 years ago and deposited at the LSHTM cryobank [4], [20], [22]. In all cases *Leishmania* were isolated from patients as part of normal diagnosis and treatment with no unnecessary invasive procedures and with written and/or verbal consent recorded at the time of clinical examination. Data on isolates were coded and anonymised. Isolation of *Leishmania* during the course of this study and not obtained from historical reference collections, whether from patients or animals, was subject to a local ethical review and approval in Paraguay (human and animal samples) by the Ethical-Scientific Committee at the IICS-UNA, under Code P42/07. All *L. infantum* and *L. donovani* strains origin from different central cryobanks (**Table S1**) and were already the object of many publications [4], [37], [39]–[41], [43].

## Results

### Genetic diversity and population structure of NW *L. infantum*

Ninety-eight strains of *L. infantum* from seven South and Central American countries with emphasis on Brazil have been studied by microsatellite analysis (**Figure 1** and **Table 1**). Most of these strains were isolated in the drier, poorly forested regions and represented human or canine isolates, but several were isolated from wild animal reservoirs, foxes (*Cerdocyon thous*) and opossums (*Didelphis marsupialis*), in the Amazonian forest region (Pará, Colombia). Isolates from different clinical forms (human VL and ACL), as well as three strains from VL/HIV co-infections were included. **Table 2** shows the variability measures for the 14 microsatellite loci in NW *L. infantum*. The number of alleles varied between 2–8, with a mean value of 5.1. The most variable markers were, as in a previous study on OW *L. infantum* [4], Lm2TG and Li 22–35. The observed heterozygosity was very low (mean *H*_o_ = 0.04) and always much lower than the expected (mean *H*_e_ = 0.216). Inbreeding coefficients varied between 0.489 and 1 (mean *F*_IS_ = 0.818). This disparity between expected and observed heterozygosity and the high *F*_IS_ values points to a considerable amount of inbreeding and/or population substructuring, reflecting possible Wahlund effect.

**Table 2:** Characterization of the 14 microsatellite markers used for population analysis of NW *L. infantum*.

Marker	Population *K*2	n	Repeat array	Fragment size array [bp]	A	*H_e_*	*H_o_*	*F*_IS_
Lm2TG	Pop1	89	TG 24–29	140–150	6	0.597	0.225	0.625
	Pop2	9	TG 20–25	132–142	3	0.391	0	1.000
	**overall**	**98**	**TG 20–29**	**132–150**	**8**	**0.652**	**0.204**	**0.688**
TubCA	Pop1	89	CA 4–9	70–80	2	0.011	0.011	0
	Pop2	9	CA 8–10	78–82	3	0.621	0.111	0.830
	**overall**	**98**	**CA 4–18**	**70–82**	**4**	**0.089**	**0.020**	**0.772**
Lm4TA	Pop1	87	TA 12–28	79–111	5	0.481	0.103	0.786
	Pop2	9	TA 10–26	75–107	3	0.627	0	1.000
	**overall**	**96**	**TA 10–28**	**75–111**	**7**	**0.567**	**0.094**	**0.835**
Li 41-56 (B)	Pop1	89	CA 9–11	88–92	3	0.033	0.011	0.665
	Pop2	9	CA 10–14	90–98	3	0.307	0.111	0.652
	**overall**	**98**	**CA 9–14**	**88–98**	**6**	**0.198**	**0.020**	**0.897**
Li 46-67 (C)	Pop1	89	CA 9	80	1	0	0	0
	Pop2	9	CA 8	78	1	0	0	0
	**overall**	**98**	**CA 8–9**	**78–80**	**2**	**0.168**	**0**	**1.000**
Li 22-35 (E)	Pop1	89	CA 5–16	78–100	4	0.066	0.022	0.661
	Pop2	9	CA 12–39	92–146	5	0.791	0.333	0.593
	**overall**	**98**	**CA 5–39**	**78–146**	**8**	**0.165**	**0.051**	**0.693**
Li 23-41 (F)	Pop1	87	GT 15–17	83–87	3	0.110	0.023	0.792
	Pop2	9	GT 2–21	57–95	3	0.627	0	1.000
	**overall**	**96**	**GT 2–21**	**57–95**	**6**	**0.266**	**0.021**	**0.922**
Li 45-24 (G)	Pop1	87	CA 15–16	105–107	2	0.034	0.011	0.664
	Pop2	9	CA 16–18	107–111	3	0.601	0	1.000
	**overall**	**96**	**CA 15–18**	**105–111**	**4**	**0.202**	**0.010**	**0.949**
Li 71-33 (P)	Pop1	88	TG 8–11	99–105	4	0.067	0.045	0.322
	Pop2	9	TG 8–11	99–105	2	0.209	0	1.000
	**overall**	**97**	**TG 8–11**	**99–105**	**4**	**0.080**	**0.041**	**0.489**
Li 71-5/2 (Q)	Pop1	89	CA 8–9	108–110	2	0.011	0.011	0
	Pop2	9	CA 6–9	104–110	4	0.706	0	1.000
	**overall**	**98**	**CA 6–9**	**104–110**	**4**	**0.108**	**0.010**	**0.906**
Li 71-7 (R)	Pop1	89	CA 12–21	98–116	4	0.044	0.022	0.496
	Pop2	9	CA 6–13	86–100	3	0.569	0.111	0.814
	**overall**	**98**	**CA 6–21**	**86–116**	**6**	**0.109**	**0.031**	**0.719**
CS20	Pop1	89	TG 18–22	83–91	2	0.022	0	1.000
	Pop2	9	TG 18–23	83–93	5	0.667	0.444	0.347
	**overall**	**98**	**TG 18–23**	**83–93**	**5**	**0.182**	**0.041**	**0.776**
LIST7031	Pop1	89	CA 10–23	109–135	3	0.044	0	1.000
	Pop2	9	CA 11–21	111–131	2	0.111	0.111	0
	**overall**	**98**	**CA 10–23**	**109–135**	**4**	**0.050**	**0.010**	**0.798**
LIST7039	Pop1	87	CA 10–15	197–207	2	0.023	0	1.000
	Pop2	9	CA 18	213	1	0	0	0
	**overall**	**96**	**CA 10–18**	**197–213**	**3**	**0.190**	**0**	**1.000**

Two main populations were inferred by STRUCTURE analysis of the 98 strains of NW *L. infantum*. The 14 microsatellite markers used for population studies were characterized for each of these two populations as well as overall for all studied strains of NW *L. infantum*. N, number of strains; A, number of alleles; *H_o_*, observed heterozygosity; *H_e_*, expected heterozygosity; *F*_IS_, inbreeding coefficient.

**Figure 2:**
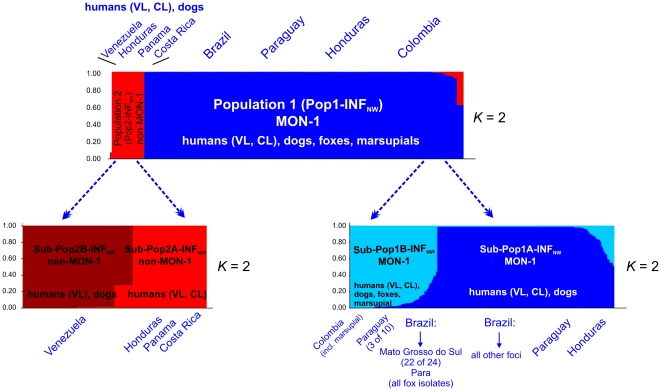
Estimated population structure and substructure of NW *L. infantum* as inferred by the STRUCTURE program. Results are based on MLMT of 14 microsatellite markers obtained for the 98 strains of NW *L. infantum* studied. In the barplots each strain is represented by a single vertical line divided into *K* colors, where *K* is the number of populations assumed. Each color represents one population, and the length of the colors segment shows the strain's estimated proportion of membership in that population. Isolates are organized by membership coefficients. According to Δ*K* the most probable number of populations in the data set is two, corresponding to MON-1 (blue) and non-MON-1 (red) isolates. In each of these main populations Δ*K* calculations suggest two sub-populations. VL – visceral leishmaniasis, CL – cutaneous leishmaniasis, CanL – canine leishmaniasis.

**Figure 3:**
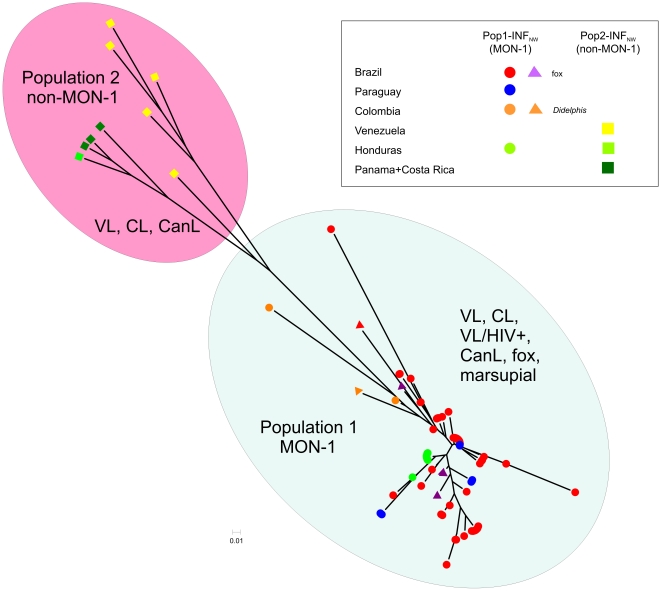
Neighbor-joining tree inferred from the Chord-distance calculated for the MLMT profiles of NW *L. infantum*. This unrooted tree was derived based on the MLMT profiles of 14 microsatellite markers for the studied sample set of 98 strains of NW *L. infantum* as used for Bayesian model-based analysis with the STRUCTURE program. The two populations inferred by STRUCTURE (*K*2) are found also by this distance –based method and are indicated on the tree. Strain origins are explained in the window beside. Marsupial and fox isolates are marked by triangles. VL – visceral leishmaniasis, CL – cutaneous leishmaniasis, CanL – canine leishmaniasis.

STRUCTURE analysis indicated that the sample set of NW *L. infantum* comprised two main populations as inferred by Δ*K* calculation (**Figure 2**). This population structure has been confirmed by distance analysis and the inferred neighbor-joining tree (**Figure 3**). Population 1 (Pop1-INF_NW_) which includes 89 of the 98 strains consists of all strains from Brazil, Paraguay and Colombia, and all but one strain from Honduras, regardless of whether they were isolated from cases of VL, VL/HIV+ or CL, or from different animal reservoirs, such as dogs and foxes. It also included the single opossum isolate. Population 2 (Pop2-INF_NW_) comprises only 9 strains, mostly from the Caribbean region (all strains from Costa Rica, Panama, Venezuela and one strain from Honduras) isolated from VL, CL and canine leishmaniasis (CanL) cases (**Table S1**). The distribution of the respective populations 1 and 2 among the Central and South American countries is shown in **Figure 1A**. *F*-statistics showed significant genetic differentiation between these two populations: *F*_ST_ = 0.761 and *p* = 0.0001.

A very important observation was that Pop1-INF_NW_ is very homogeneous with only 35 genotypes identified for the 89 strains (proportion of polymorphic strains in pop1 = 39%), whereas in Pop2-INF_NW_ all strains had unique genotypes. **Table 3** gives a detailed overview of the number of genotypes found in each country studied and **Figure S1** shows a neighbor-joining tree, which is based only on distinct genotypes. Interestingly, 13 out of the 15 Honduran strains in Pop1-INF_NW_ including all CL cases, shared an identical MLMT profile. Another cluster of identical MLMT profiles was found for eight strains of human and canine origin from Mato Grosso do Sul. A third big cluster of identical genotypes comprised 23 strains from Espírito Santo, Rio de Janeiro, Pernambuco, Ceará, and three from Paraguay. Also here an identical MLMT profile was shared by human VL, VL/HIV+ and canine isolates (**Figure S1**, **Figure S2**). The difference in the degree of polymorphism between Pop1-INF_NW_ and Pop2-INF_NW_ is also reflected by the mean number of alleles (MNA) of 3.1 for population 1 (N = 89) and of 2.9 for population 2 (N = 9) (**Table 4**). In both populations the observed heterozygosity was much lower than the expected, leading to high *F*_IS_ values (**Tables 2** **and** **4**).

**Table 3:** Number of MLMT genotypes found for each country and the studied endemic regions of Brazil.

Country	Region	No. of strains	No. of MLMT genotypes
Costa Rica		1	1
Panama		2	2
Honduras		15	3
Venezuela		5	5
Colombia		3	3
Paraguay		10	3
Brazil		62	36
	*Rio de Janeiro*	*3*	*1*
	*Espírito Santo*	*10*	*5*
	*Ceará*	*5*	*2*
	*Bahia*	*3*	*3*
	*Pará*	*5*	*4*
	*Rio Grande do Norte*	*2*	*2*
	*Pernambuco*	*4*	*3*
	*Piauí*	*3*	*3*
	*Mato Grosso do Sul*	*24*	*13*
	nd	*3*	*2*

**Table 4:** Characterization of the two main populations found for the analysed 98 NW *L. infantum* strains.

Population	Origin	N	P	MNA	*H*_o_	*H*_e_	*F*_iS_
Pop1-INF_NW_	Brazil, Paraguay, Honduras, Colombia	89	0.786	3.1	0.035	0.110	0.676
Pop2-INF_NW_	Venezuela, Honduras, Panama, Costa Rica	9	0.857	2.9	0.087	0.445	0.813
overall		98	0.821	5.1	0.040	0.216	0.818

N, number of strains; P, proportion of polymorphic loci; MNA, mean number of alleles; *H_o_*, observed heterozygosity; *H_e_*, expected heterozygosity; *F*_IS_, inbreeding coefficient.

Both main populations have been tested by STRUCTURE for sub-structures. Two sub-populations were found for each of the main populations (**Figure 2**). Sub-Pop1B-INF_NW_ (34 strains) comprised 22 of the 24 strains from Mato Grosso do Sul, all fox isolates from Pará, all Colombian strains including the *D. marsupialis* isolate, and three canine strains from Paraguay. The other 55 strains were members of Sub-Pop1A-INF_NW_ consisting of all strains from Honduras, seven strains from Paraguay and the strains from all other Brazilian foci. Both 1A and 1B sub-populations included strains isolated from all clinical forms of human disease as well as from dogs. Population 2 was divided into Sub-Pop2B-INF_NW_ comprising all Venezuelan strains isolated from human cases of VL and CL and from dogs, and Sub-Pop2A-INF_NW_ consisting of the CL strains from Panama and Costa Rica, and a single VL strain from Honduras. Distance analysis confirmed the inferred subpopulations (**Figures S1 and S2**). *F*_ST_ analysis however showed that genetic differentiation of the sub-populations was statistically not significant. The assignment of the strains to the respective populations and sub-populations is given in **Table S1**.

### Combined population analysis of NW and OW *L. infantum*

To address questions about the nomenclature and the origin of NW *L.infantum* we have included in the analysis previously identified MLMT profiles of 308 *L. infantum* strains from different countries of southern Europe, North Africa, the Middle East and Asia and, as an outgroup, 20 *L. donovani* strains from East Africa and India [4], [37], [39]–[41], [43] (**Table S1**, **Table 1**). Most of the *L. infantum* strains belong to the zymodeme MON-1, the most ubiquitous in the Old World, however several non-MON-1 strains have been also included. All these strains represented different clinical pictures in humans, namely VL, CL, VL/HIV+, PKDL, as well as canine, fox and sand fly isolates (**Table 1**).

**Figure 4:**
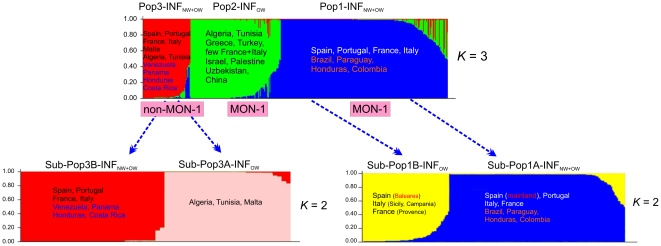
Estimated population structure and substructure of the combined New World and Old World sample set. MLMT profiles based on 14 microsatellite markers for 308 OW strains and 98 NW strains of *L. infantum* were analysed by Bayesian statistics implemented in the STRUCTURE software. In the barplots each strain is represented by a single vertical line divided into *K* colors, where *K* is the number of populations assumed. Each color represents one population, and the length of the colors segment shows the strain's estimated proportion of membership in that population. Isolates are organized by membership coefficients. According to Δ*K* the most probable number of populations in the data set is three, corresponding to MON-1 isolates from Southwest Europe and the New World (blue), MON-1 isolates from North Africa, Southeast Europe and Asia (green), and non-MON-1 islates from the Old and New World (red). Δ*K* calculations suggest two sub-populations in the main populations Pop1 and Pop3.

STRUCTURE analysis of the combined NW and OW *L. infantum* strains revealed 3 main populations, as deduced from Δ*K* calculation (**Figure 4**), which showed significant genetic differentiation (**Table 5**). The largest population (Pop1-INF_NW+OW_) was formed by 224 strains from Spain, Portugal, France, Italy (all identified as MON-1) as well as from Brazil, Paraguay, Honduras (all but one strain) and Colombia. The second population (Pop2-INF_OW_) comprised 121 MON-1 strains from Algeria, Tunisia, Greece, Turkey, Israel, Palestine, Uzbekistan, China, and few from France and Italy. Strains from the New World were not found in this population. The third population (Pop3-INF_NW+OW_) consisted of 59 strains from southwestern Europe and North Africa (all identified as non-MON-1) as well as from Venezuela, Panama, Costa Rica and a single strain from Honduras. Consequently, population 1 of NW *L. infantum* (Pop1-INF_NW_) is part of the southwestern European *L. infantum* MON-1 population, and population 2 of NW *L. infantum* (Pop2-INF_NW_) of the Mediterranean non-MON-1 *L. infantum* population. The non-MON-1 population was the most variable one, as shown by the highest values for the MNA (9.6), *H*_o_ (0.27) and *H*_e_ (0.777), although the sample size was much smaller than that of the other two populations. This is in agreement with previous observations for Mediterranean *L. infantum* [4]. All three populations showed high *F*_IS_ values, especially the two MON-1 populations (**Table 6**).

**Table 5:** *F*_ST_ values and corresponding *p*-values for the main three populations.

*F*_ST_-values:	Population 1 INF_NW+OW_	Population 2 INF_OW_	Population 3 INF_NW+OW_
Pop1- INF_NW+OW_ MON-1 (224)	0	0.376	0.459
Pop2-INF_OW_ MON-1 (121)	0.0003	0	0.277
Pop3-INF_NW+OW_ non-MON-1 (59)	0.0003	0.0003	0

Data are based on the combined analysis of NW and OW *L. infantum* and the populations inferred by STRUCTURE. *F*_ST_ values are in the upper triangle, *p*-values in the lower triangle. Number of strains in parentheses. Without ES9III and ES10III (mixed genotypes/hybrids).

**Table 6:** Characterization of the main populations found by STRUCTURE analysis for the 406 *L. infantum* strains.

Population	Origin	N	P	MNA	*H*_o_	*H*_e_	*F*_IS_
Pop1-INF_NW+OW_	Brazil, Paraguay, Honduras, ColombiaSpain, Portugal, France, Italy	224	0.929	6.5	0.034	0.231	0.853
Pop2-INF_OW_	Algeria, Tunisia, Greece, Turkey, China,Uzbekistan, Israel, Palestine, few France+Italy	121	1.000	5.4	0.071	0.420	0.832
Pop3-INF_NW+OW_	Venezuela, Panama, Honduras, Costa RicaSpain, Portugal, France, Italy, MaltaAlgeria, Tunisia	61	1.000	9.6	0.272	0.777	0.651

N, number of strains; P, proportion of polymorphic loci; MNA, mean number of alleles; *H_o_*, observed heterozygosity; *H_e_*, expected heterozygosity; *F*_IS_, inbreeding coefficient.

The two main populations which included the NW *L. infantum* strains were tested for sub-structures. Population 1 (Pop1-INF_NW+OW_) was clearly divided into two sub-populations, as inferred by Δ*K* (**Figure 4**), with significant genetic differentiation between them (*F*_ST_ = 0.223, *p* = 0.0001). Sub-Pop1A-INF_NW+OW_ (N = 149 strains) comprised the majority of strains and included strains from the Iberian mainland, Italy, and France as well as the NW strains. Sub-Pop1B-INF_OW_ (N = 75 strains) contained all Balearic strains, strains from Sicily and Campania (Italy) and from the Provence region of France. The sub-structures of the non-MON-1 population 3 (Pop3-INF_NW+OW_) were not very clear, however a first split led to Sub-Pop3A-INF_OW_ formed by 28 strains from North Africa and Malta. Sub-Pop3B-INF_NW+OW_ comprised 31 strains from Europe and the NW (**Figure 4**). There is significant genetic differentiation between these sub-populations (*F*_ST_ = 0.25, *p* = 0.0001). **Table 7** presents the variability measures for each of the detected sub-populations. The most homogeneous population was Sub-Pop1A-INF_NW+OW_, which comprised *L. infantum* MON-1 (MNA = 3.3), even though its sample size was the largest. High *F*_IS_ values were detected for each of these sub-populations.

**Table 7:** Characterization of sub-populations of populations 1 and 3 of combined NW and OW *L. infantum*.

Sub-Population	Origin	N	P	MNA	*H*_o_	*H*_e_	*F*_IS_
Sub-Pop1A-INF_NW+OW_	Brazil, Paraguay, Honduras, ColombiaSpain (mainland), Portugal, France, Italy	149	0.714	3.3	0.023	0.135	0.829
Sub-Pop1B-INF_OW_	Spain (Baleares), Italy (Sicily, Campania),France (Provence)	75	1.000	5.9	0.056	0.343	0.839
Sub-Pop3A-INF_OW_	Algeria, Tunisia, Malta	28	1.000	6.5	0.414	0.715	0.426
Sub-Pop3B-INF_NW+OW_	Venezuela, Panama, Honduras, Costa RicaSpain, Portugal, France, Italy	31	1.000	6.4	0.118	0.613	0.811

Populations and sub-populations were inferred by STRUCTURE analysis. N, number of strains; P, proportion of polymorphic loci; MNA, mean number of alleles; *H_o_*, observed heterozygosity; *H_e_*, expected heterozygosity; *F*_IS_, inbreeding coefficient; FR – France; IT – Italy.

**Figure 5:**
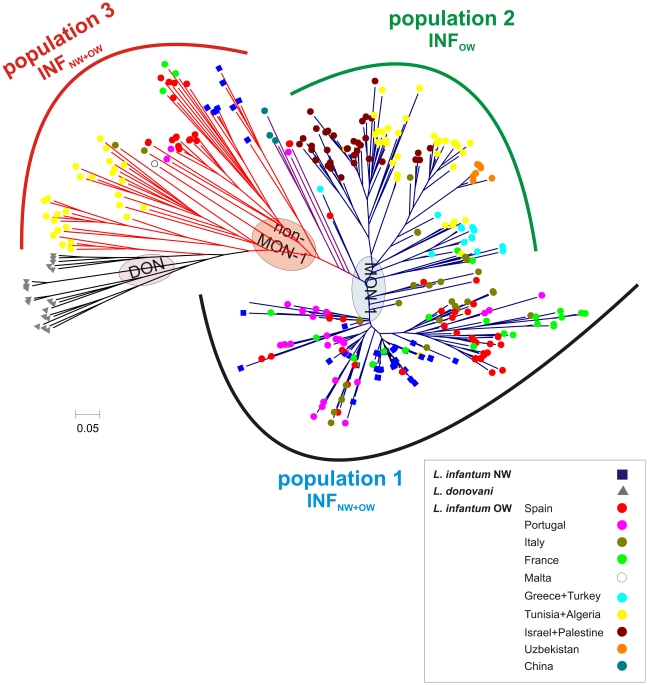
Neighbor-joining tree derived for the combined New World and Old World data set. This unrooted tree was inferred from the Chord-distance calculated for the MLMT profiles of 14 microsatellite markers for the studied sample set of 98 NW strains of *L. infantum* (marked by blue squares), 308 OW strains of *L. infantum* (marked by dots of different colors, depending on their origin) and 20 strains of *L. donovani* (marked by grey triangles). The three populations inferred by Bayesian model-based analysis with the STRUCTURE program (*K*3) and shown in Fig. 4 are indicated. MON-1 strains are marked by blue branches, non-MON-2 strains by red branches, and *L. donovani* by black branches. NW strains of *L. infantum* are present in population 1 and 3. Strains isolated from *Didelphis marsupialis* and *Cerdocyon thous* are interspersed among population1 that included the majority of NW strains (human and canine) together with strains of *L. infantum* from the Iberian mainland (Spain, Portugal), France and Italy. Strain origins are listed in the legend beside.

The same clustering and population structure was found by distance analysis of the MS data. **Figure 5** shows the neighbor-joining tree inferred from the combined sample set of 426 strains from the Old and New World with *L. donovani* as outgroup. NW strains of *L. infantum* are intermingled among the European *L. infantum* MON-1 and non-MON-1 strains. Identical genotypes were found multiple times for strains of NW and OW *L. infantum* in population 1 (Pop1-INF_NW+OW_), e.g. between strains from Mato Grosso do Sul (Brazil) and a strain from Portugal, between strains from eastern Brazil, Paraguay, and from France and Spain. Furthermore, one of the Honduran VL strains was identical to strains from Portugal and France (data not shown). NW strains of *L. infantum* were concentrated in few major clusters that also contained strains from the Iberian peninsula and France, indicating an expansion of single clones (**Figure S3**, **Figure 5**).

## Discussion

### Population structure of NW *L. infantum* and comparison with OW *L. infantum* population structure

Knowledge of the population structure of NW *L. infantum* was so far very restricted and essentially based on clinical observations and the population structure of the vector. With the development of MLMT an adequate and powerful tool based on highly polymorphic genetic markers that differentiate at intra-species level became available for analysing population structure of the parasite. The present paper shows that there are two main populations of NW *L. infantum*, that correlate with the separation of *L. infantum* into MON-1 and non-MON-1 strains [4], [43]. Ninety-one per cent of the 98 NW strains analysed fell into the MON-1 population (Pop1-INF_NW_), which extends over a huge geographical and ecological range, including strains from all Brazilian, Paraguayan and some Colombian foci, and from Honduras. This situation is very similar to that in the OW, where ∼70% of *L. infantum* strains isolated in foci in southern Europe, Middle East, Central Asia and North Africa belong to zymodeme MON-1 [53]. The remaining 9% of the NW *L. infantum* strains belong to the non-MON-1 population 2 (Pop2-INF_NW_) and, interestingly, all these strains come from the Caribbean region, from Panama, Costa Rica and Venezuela, and a single strain from Honduras. Whether the non-MON-1 strains are generally present exclusively in the Caribbean region needs to be further elucidated.

The focal point of our sampling was Brazil, including most of the known foci. Although Brazil is an ecologically diverse country and different reservoirs and vector populations have been reported, all the 64 Brazilian strains from different VL foci belonged to the MON-1 population 1. The same was true for the neighboring country Paraguay.

The degree of polymorphism in NW *L. infantum* is much lower than among OW *L. infantum*. Only 39% of the NW MON-1 strains had individual MLMT profiles compared to about 75% of the OW MON-1 strains analysed in this and in a previous study [4]. The lower variability of NW *L.infantum* is also reflected by lower MNA and *H*_e_ values for both the MON-1 and non-MON-1 populations (**Table 4** **and Table S2**). Moreover, in contrast to the NW genetically clearly separated geographically determined sub-populations were observed in the OW MON-1 cluster. The lower diversity of NW *L. infantum* supports the hypothesis of a recent import of selected strains of *L. infantum* from the Old to the New World [54]. In the case of indigenous parasites we would expect a much higher diversity and more complex population structures.

Despite the low diversity of NW *L.infantum* we found indications for population sub-structures. The MON-1 sub-populations are mostly geographically determined. Two sub-populations were recognized in Brazil and Paraguay, Sub-Pop1B-INF_NW_ comprised three strains from central Paraguay, all but two strains from the Mato Grosso do Sul focus and those from rural foci in Pará. All other strains were members of the Sub-Pop1A-INF_NW_. Strains from Colombia and Honduras belonged to the sub-populations 1B and 1A, respectively. There is some hint that the Colombian strains may form a separate population, but this has to be proven with more strains from that country.

Three big clusters were detected that contain genetically identical strains. The first two included strains from the same focus, eight strains from Mato Grosso do Sul and 13 from Honduras, respectively. The third comprised 26 strains from seven states of Brazil, mostly from the eastern part of the country and some from Paraguay. This is evidence for the spread of several single clones in the New World. The homogeneity of strains in Honduras where only three genotypes were identified for 15 strains collected between 1987 and 1994 was surprising and needs further investigation.

The strong heterozygote deficiency and high inbreeding coefficients found for NW and OW *L. infantum* could result from population subdivision (Wahlund effect), presence of null alleles, natural selection, gene conversion, and inbreeding. Since similar *F*_IS_ patterns were obtained across all 14 microsatellite loci in this study, null alleles, selection and gene conversion are unlikely to be responsible for the heterozygote deficiency found. Recent studies [55], [56] have demonstrated the existence of Wahlund effect in *Leishmania* populations which did however account only partially for the high inbreeding and suggested the “existence of population foci at a microgeographic scale, where clonality alternates with sexuality of an endogamic nature”. The strains of NW *L. infantum* analysed herein were sampled over large areas and further geographic subdivision of the subpopulations identified seems to be quite likely. For clarifying whether the high inbreeding is exclusively due to population subdivision or also to the presence of sexual recombination, mainly among identical or similar organisms, sampling at finer geographical scale would be essential.

### Clinical picture, reservoirs and vectors versus NW *L. infantum* populations and sub-populations

There was no strict correlation between clinical pictures and population assignment. Strains isolated from human VL and CL cases, including the atypical CL cases collected exclusively in the Caribbean, were assigned to both the MON-1 and non-MON-1 populations. VL/HIV+ coinfections were found only in the MON-1 population, but due to the small number of these samples so far analysed we cannot draw conclusions. In Pop1-INF_NW_ strains from CL cases from Honduras formed a single cluster of identical genotypes and one of the two VL strains from Honduras was similar to this CL cluster. This confirms previous reports showing by kDNA RFLP and RAPD that the two clinical forms in Honduras and Nicaragua were caused by genetically similar parasites [20], [21]. In addition, we found that at least two different parasite populations circulate in Honduras. Disease susceptibility and clinical manifestation is, however not only affected by parasite factors but also by host conditions, such as malnutrition and the age of the patient (status of the host immune system). In Honduras VL patients were much younger than CL patients. The vector may also play a role, as two species, *Lu. longipalpis* and *Lu. evansi*, are present in the Honduran endemic foci.

Obviously, we cannot link the restricted occurrence of CL cases in the Caribbean region with the general population assignment to MON-1 and non-MON-1 strains. From the Old World it is known, that only 20% of the CL cases are due to MON-1 strains and that the majority of CL cases is caused by strains of zymodeme MON-24, besides other dermotropic zymodemes, some of which were represented by several strains in this study. Ninety percent of all VL cases of immunocompetent individuals are caused by MON-1 strains, but there are also several other viscerotropic zymodemes [57], [58]. The tropism of many zymodemes is not clear cut, they are known to cause both VL and CL and the reasons leading to the respective clinical picture seem to be very complex.

The dog is the main reservoir host for NW *L. infantum* [59] and this was confirmed by the detection of identical MLMT profiles for human and canine strains from different VL foci, such as Mato Grosso do Sul, Ceará, Espírito Santo, Rio de Janeiro (Brazil) and Paraguay. All canine strains were found in the MON-1 population except one from Venezuela which was linked to the non-MON-1 population. This is in agreement with the situation in the OW where the majority of canine leishmaniasis is due to strains of zymodeme MON-1 [58], [60], [61]. Only very few other zymodemes, MON-98, 77, and 108, which are closely related to MON-1, and MON-253 and MON-24 have occasionally been found in dogs.

It has been suggested that natural foci of sylvatic zoonotic transmission may exist beside the main transmission cycle via the domestic dog [62]–[64]. Foxes are considered to be a natural reservoir of VL in different states of Brazil, such as Pará [65], Mato Grosso do Sul [66], Ceará and Piauí [63], Minas Gerais [67], [68], and Amazonas [69]. *Didelphis marsupialis* has been incriminated as an important reservoir host of NW *L. infantum* only in Colombia [70]–[72]. Opossums infected by NW *L. infantum* have, however also been reported from Bahia and Minas Gerais [73], [74]. The role of these animals in natural infection cycles remains however, questionable [73], [75]. MLMT analysis did not reveal separate genotypes for strains from wild animals. The four isolates from foxes and the one from a marsupial were all assigned to the MON-1 Sub-Pop1B-INF_NW_ and interspersed with the human and canine strains (**Figure 2**, **Figure 3** and **Figures S1**, **S2**, **S33**).

The transmission of NW *L. infantum*, its virulence and clinical picture are likely influenced by coevolutionary interactions between specific parasite and sand fly genotypes, as suggested recently [26], and different sand fly species or subspecies might be involved in the transmission of different *L. infantum* genotypes in the NW. *Lu. longipalpis*, the major vector of NW *L. infantum*, is distributed from Southern Mexico to Northern Argentina and it is considered to be a complex of sibling species [15], [24]–[26], [28]. Arrivillaga et al. (2002) have concluded from mitochondrial sequence (COI) data that *Lu. longipalpis* in Central and South America consists of at least four clades, which constitute species [27]. These clades may correlate in part with some of the populations or subpopulations of NW *L. infantum* identified in this study.

The Caribbean non-MON-1 population might possibly be linked to *Lu. evansi* which was reported as an alternative vector for NW *L. infantum* in Latin America [15], but was not found in Brazil. Interestingly, most strains from Mato Grosso do Sul and three from Paraguay were found in Sub-Pop1B-INF_NW_ whereas the strains from all other Brazilian and Paraguyan foci were assigned to Sub-Pop1A-INF_NW_. This could be attributed to the fact that in some foci of Mato Grosso do Sul *Lu. cruzi* (a species within the *Lu. longipalpis* complex) has been established as the vector for NW *L. infantum* and it has been found to be sympatric with *Lu. longipalpis* in many areas [76]–[82]. It is perhaps also present in bordering areas of Paraguay and Bolivia [77], [79], [80], [83]. Whether different geographically determined *L. infantum* genotypes correlate with the occurrence of specific sand fly species in those areas should be further investigated.

### Origin of NW *L. infantum*

**Figure 6:**
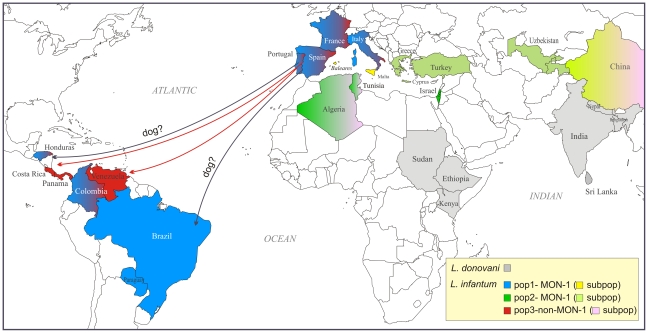
Map showing the presumable origin of NW *L. infantum*. The respective populations of *L. infantum* in the Old and New Worlds are indicated by the respective colors used in Fig. 2 and 5. NW *L. infantum* has been introduced from Southwest Europe to the New World multiple times.

Our results indicate that *L. infantum* was introduced from Southwest Europe to the New World several times and at several points along this continent (**Figure 6**). The genotypes found in specific regions in South and Central America were also found in Europe, especially among the Spanish, Portuguese, French and Italian strains of *L. infantum*. When analysed together the MON-1 population of NW *L. infantum* was intermingled with MON-1 strains from the Western European Mediterranean countries and the non-MON-1 population comprised strains from both New and Old Worlds.

There were several waves of immigration of Europeans into the New World especially from *Leishmania* endemic countries such as Portugal and Spain starting with the arrival of the Conquistadores up to the immigration of settlers during last century. Brought to South America with European immigrants, perhaps many times, the parasites spread rapidly due to migration, urbanization and trade. There is e.g. some indication of expansion of single genotypes (clones) among the Northeast of Brazil and Mato Grosso do Sul, respectively. This introduction of *L. infantum* is still ongoing as recently shown for an infected dog which was brought from France to French Guiana [17].

This study further confirmed that the agent of VL in the New World is *L. infantum* and not a separate indigenous species, *L. chagasi*. This is also supported by i) the identity of the isoenzyme profiles [44], [54], [84]–[88], ii) the identical genotypes obtained by analysing different genomic targets with different molecular techniques [2]–[4], [38], [43], [89]–[92], iii) the fact that *L. infantum* can be transmitted by several OW sand fly species and infect, develop in and adapt to *Lu. longipalpis* [32], and (iv) by the fact that New World foxes are phylogenetically closer to Old World wolf-like canids than to Old World foxes and therefore have a high potential to be a reservoir [93]. It still remains unclear, whether neotropical wild animals found to be infected with *L. infantum* are accidental hosts or real reservoirs. We could show, that parasites isolated from four foxes and one marsupial did not constitute a separate population in the NW and are, thus, not part of separate transmission cycles.

Multiple introductions of the parasite help to explain the immense spread of *L. infantum* in the NW Furthermore, *Lu. longipalpis* has been proven to be a permissive vector and fast adaptation is facilitated by modification of the parasite's surface molecules [94], [95]. Thus *L. infantum* brought to the NW could have easily adapted to the respective local sand fly populations.

Recent, and likely continious migrations to and possibly even from, the NW are further supported by *F*_ST_ values calculated between NW and OW *L. infantum*, which indicate only little genetic differentiation (data not shown). In contrast, we observed that the populations of *L. infantum* from southern Europe are more closely related to NW *L. infantum* than to other populations of OW *L. infantum*, e.g. from North Africa, Central Asia, the Middle East and even South Eastern Europe. Moreover, parasites indigenous in the NW should be more diverse, but we observed them to be much less diverse than *L. infantum* or *L. donovani* from the Old World. This is consistent with a founder effect, i.e. recent introduction of a restricted part of the original *L. infantum* population, with possible genetic drift or clonal expansion of only some genotypes. As a consequence there is no justification for a taxonomic separation of *L. chagasi* and *L. infantum* at species or subspecies level .

The present paper represents to our knowledge the first comprehensive population study of NW *L .infantum*, in which we have applied a high resolution typing method sensitive enough to detect population structures at intra-species level. We found a very homogenous population structure in Brazil and Paraguay consisting exclusively of MON-1 strains and a mixed population structure including MON-1 and non-MON-1 strains in the Caribbean region. Further studies with refined sampling strategies based on the populations and sub-populations detected in this study will enable more intensive microepidemiological analyses of NW *L. infantum* genotypes, and their association with reservoirs, vectors, clinical presentation, host immunological status, ecology, geography, and socioeconomic or demographic factors. We have provided conclusive evidence of recent multiple introductions of *L. infantum* from the Old into the New World including both MON-1 and non-MON-1 genotypes and for the synonymy of *L. infantum* and *L. chagasi*.

## Supporting Information

Figure S1 **Neighbor-joining tree of NW** ***L. infantum*** **genotypes.** This midpoint-rooted tree was derived from the Chord-distance calculated for the MLMT profiles of 14 microsatellite markers. Only different genotypes are scored. Clinical pictures, reservoir, origin and number of strains sharing the same genotype are indicated, as well as population and sub-population assignements according to Bayesian model-based analysis with the STRUCTURE program (see Fig. 2). MS - Mato Grosso do Sul, PA - Pará, RJ - Rio de Janeiro, ES - Espírito Santo, BA - Bahia, RN - Rio Grande do Norte, PI - Piauí, PE - Pernambuco, nd - not defined. VL - visceral leishmaniasis, CL - cutaneous leishmaniasis, CanL - canine leishmaniasis.
(TIF)

Figure S2 **Midpoint-rooted neighbor-joining tree showing all individual NW** ***L. infantum*** **strains.** Phylogenetic tree derived from the Chord-distance calculated for the MLMT profiles of 14 microsatellite markers for the 98 NW strains of *L.infantum* studied. Clinical pictures and reservoir for each strain are indicated. The origin of the Brazilian strains (state) is given as abbreviation: MS - Mato Grosso do Sul, PA - Pará, RJ - Rio de Janeiro, ES - Espírito Santo, BA - Bahia, RN - Rio Grande do Norte, PI - Piauí, PE - Pernambuco. Populations and sub-populations according to STRUCTURE analysis (see Fig. 2) are shown. ^T^ -WHO reference strain of NW *L. infantum* (PP75). VL – visceral leishmaniasis, CL – cutaneous leishmaniasis, CanL – canine leishmaniasis, nd – not determined.
(TIF)

Figure S3 **Neighbor-joining tree showing only population 1 of the combined NW and OW strains of** ***L. infantum*** **.** This unrooted tree is based on the Chord-distance of the MLMT profiles of 14 microsatellite markers and it includes only strains belonging to population1 (MON-1) of the combined OW (dots) and NW (squares) *L. infantum* data set (see Fig. 5 and 6). Isolates of wild animal reservoirs are indicated by arrows. NW *L. infantum* strains (all marked by blue squares) are concentrated in few clusters marked in grey, that also contain *L. infantum* from Portugal (pink dots), Spain (red dots) and France (green dots). Origins of the strains are indicated in the legend beside.
(TIF)

Table S1 b^T^ – Reference strain of the species; 1 – zymdemes according to the Montpellier system – MON [86] or the CLIOC system – IOC/Z [44], MON-1 and Z1 are corresponding zymodemes; ^2^ – Population assignment according to STRUCTURE analysis of the combined dataset of 409 strains of NW and OW *L. infantum*; ^3^ – Population assignment according to STRUCTURE analysis of the dataset of 98 NW *L. infantum* strains; - VL – visceral leishmaniasis; CL – cutaneous leishmaniasis; PKDL – post Kala-Azar dermal leishmaniasis; CanL – canine leishmaniasis; cIL – central Israel, nIL – north Israel; nd – not defined; na – not applicable; CNRFV - BIOMED-Centro Nacional de Referencia de Flebotomos de Venezuela; SENEPA - Programa Nacional de Leishmaniosis, SENEPA, Ministry of Public Health, Paraguay; CLIOC - Coleção de Leishmania do Instituto Oswaldo Cruz, Brazil; LSHTM - London School of Hygiene and Tropical Medicine, UK; KIT - Royal Tropical Institute, Amsterdam, Netherlands; CNRLM - Centre National de Référence des *Leishmania*, Université Montpellier, France; ISCM - WHO Collaborating Centre for Leishmaniasis, Servicio de Parasitología, Instituto de Salud Carlos III, Mahadahonda (Madrid), Spain; ISS - Instituto Superiore di Sanità, Italy;.HPI - Hellenic Pasteur Institute, Athens, Greece; IHMT - Instituto de Higiene e Medicina Tropical, Universidade Nova de Lisboa, Portugal; JRCL - WHO's Jerusalem Reference Centre for Leishmaniases, Hebrew University, Hadassah Medical School, Jerusalem, Israel; AQNHI - Al-Quds Nutrition and Health Research Institute, Faculty of Medicine, Al-Quds University, Abu-Deis, Palestine; IPA - Institut Pasteur d'Algérie, Algiers, Algeria; LPMM - Laboratoire de Parasitologie_Mycologie à la Faculté de Pharmacie, Monastir, Tunisia; BHU - Kala-azar Medical Research Centre, Banaras Hindu University, Varanasi, India.
(DOC)

Table S2 N, number of strains; P, proportion of polymorphic loci; MNA, mean number of alleles; *H_o_*, observed heterozygosity; *H_e_*, expected heterozygosity; *F*_IS_, inbreeding coefficient; FR – France; IT – Italy.
(DOC)

Methods S1 **Additional information about the methods of MLMT data analyses.**
(DOC)
