# Supplementary figures and images for: Comparative Microsatellite Typing of New World Leishmania infantum Reveals Low Heterogeneity among Populations and Its Recent Old World Origin
Source: PLoS Negl Trop Dis. 2011 Jun 7;5(6):e1155. doi: 10.1371/journal.pntd.0001155 (PMC3110170; doi:10.1371/journal.pntd.0001155)

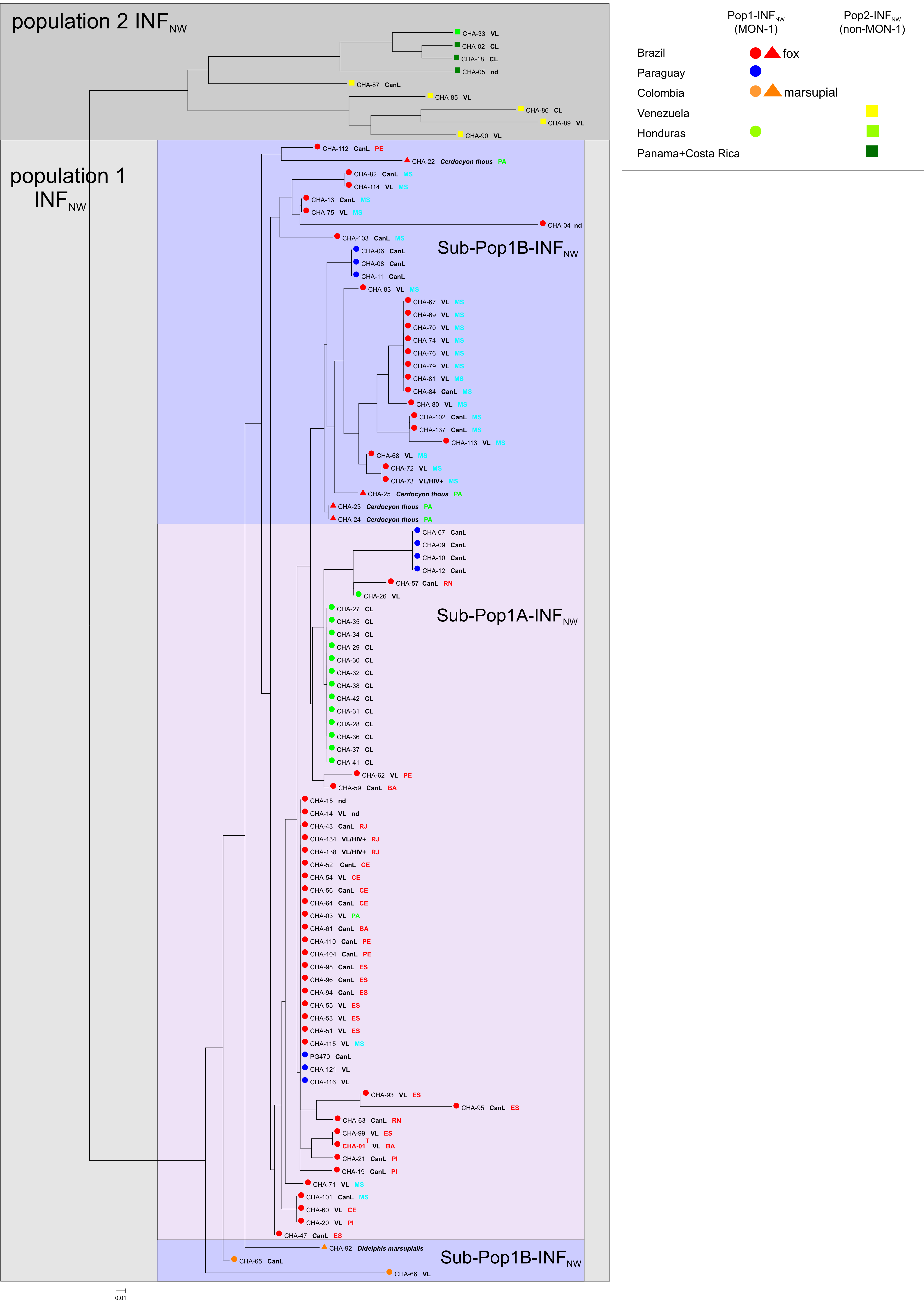

Supplement: Figure S2 — Midpoint-rooted neighbor-joining tree showing all individual NW L. infantum strains. Phylogenetic tree derived from the Chord-distance calculated for the MLMT profiles of 14 microsatellite markers for the 98 NW strains of L.infantum studied. Clinical pictures and reservoir for each strain are indicated. The origin of the Brazilian strains (state) is given as abbreviation: MS - Mato Grosso do Sul, PA - Pará, RJ - Rio de Janeiro, ES - Espírito Santo, BA - Bahia, RN - Rio Grande do Norte, PI - Piauí, PE - Pernambuco. Populations and sub-populations according to STRUCTURE analysis (see Fig. 2) are shown. T -WHO reference strain of NW L. infantum (PP75). VL – visceral leishmaniasis, CL – cutaneous leishmaniasis, CanL – canine leishmaniasis, nd – not determined. (TIF) [file pntd.0001155.s002.tif]

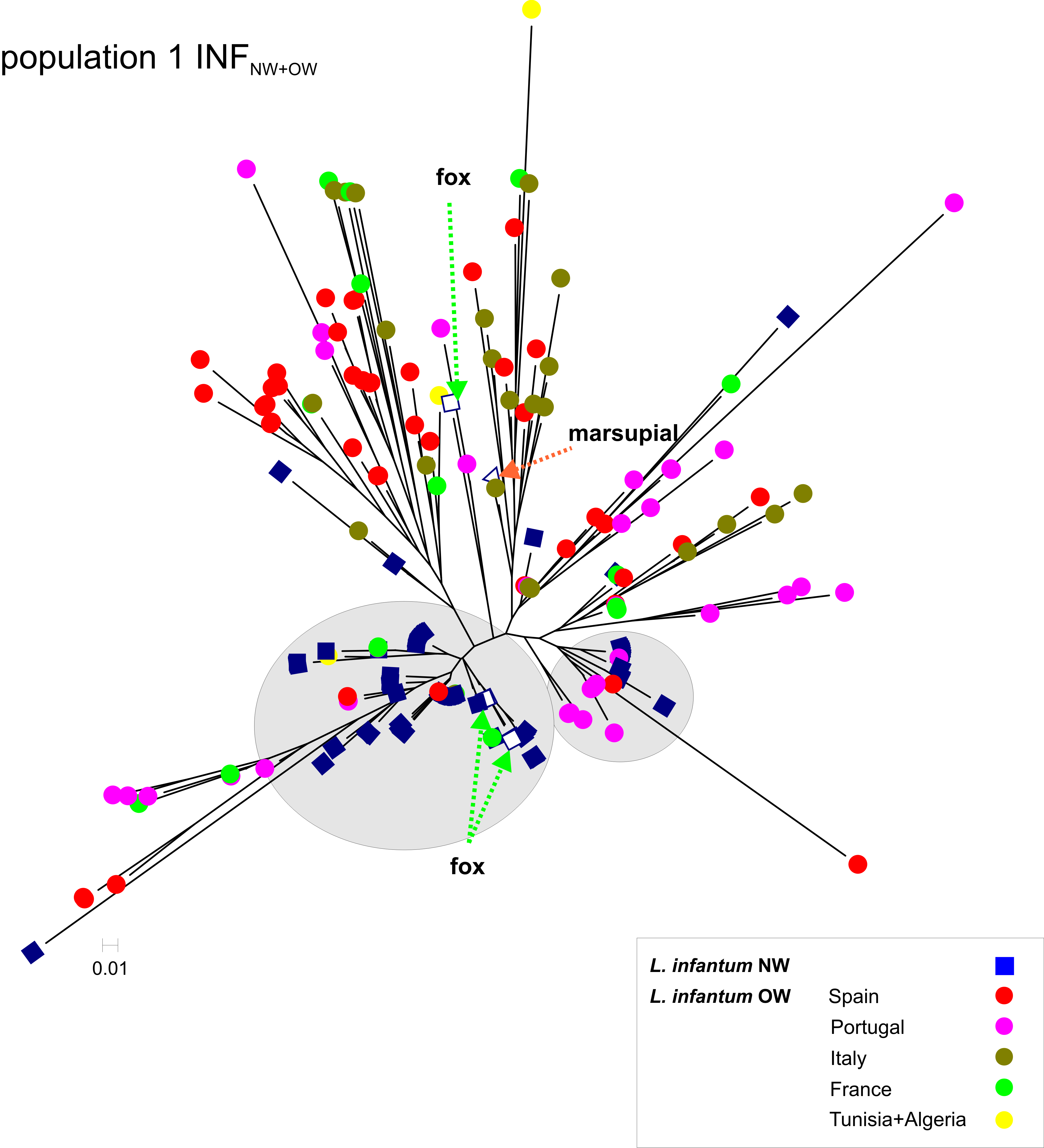

Supplement: Figure S3 — Neighbor-joining tree showing only population 1 of the combined NW and OW strains of L. infantum . This unrooted tree is based on the Chord-distance of the MLMT profiles of 14 microsatellite markers and it includes only strains belonging to population1 (MON-1) of the combined OW (dots) and NW (squares) L. infantum data set (see Fig. 5 and 6). Isolates of wild animal reservoirs are indicated by arrows. NW L. infantum strains (all marked by blue squares) are concentrated in few clusters marked in grey, that also contain L. infantum from Portugal (pink dots), Spain (red dots) and France (green dots). Origins of the strains are indicated in the legend beside. (TIF) [file pntd.0001155.s003.tif]
