# Supplementary material for: Comparative Microsatellite Typing of New World Leishmania infantum Reveals Low Heterogeneity among Populations and Its Recent Old World Origin
Source: PLoS Negl Trop Dis. 2011 Jun 7;5(6):e1155. doi: 10.1371/journal.pntd.0001155 (PMC3110170; doi:10.1371/journal.pntd.0001155)
Supplement: Table S1 — bT – Reference strain of the species; 1 – zymdemes according to the Montpellier system – MON [86] or the CLIOC system – IOC/Z [44], MON-1 and Z1 are corresponding zymodemes; 2 – Population assignment according to STRUCTURE analysis of the combined dataset of 409 strains of NW and OW L. infantum; 3 – Population assignment according to STRUCTURE analysis of the dataset of 98 NW L. infantum strains; - VL – visceral leishmaniasis; CL – cutaneous leishmaniasis; PKDL – post Kala-Azar dermal leishmaniasis; CanL – canine leishmaniasis; cIL – central Israel, nIL – north Israel; nd – not defined; na – not applicable; CNRFV - BIOMED-Centro Nacional de Referencia de Flebotomos de Venezuela; SENEPA - Programa Nacional de Leishmaniosis, SENEPA, Ministry of Public Health, Paraguay; CLIOC - Coleção de Leishmania do Instituto Oswaldo Cruz, Brazil; LSHTM - London School of Hygiene and Tropical Medicine, UK; KIT - Royal Tropical Institute, Amsterdam, Netherlands; CNRLM - Centre National de Référence des Leishmania, Université Montpellier, France; ISCM - WHO Collaborating Centre for Leishmaniasis, Servicio de Parasitología, Instituto de Salud Carlos III, Mahadahonda (Madrid), Spain; ISS - Instituto Superiore di Sanità, Italy;.HPI - Hellenic Pasteur Institute, Athens, Greece; IHMT - Instituto de Higiene e Medicina Tropical, Universidade Nova de Lisboa, Portugal; JRCL - WHO's Jerusalem Reference Centre for Leishmaniases, Hebrew University, Hadassah Medical School, Jerusalem, Israel; AQNHI - Al-Quds Nutrition and Health Research Institute, Faculty of Medicine, Al-Quds University, Abu-Deis, Palestine; IPA - Institut Pasteur d'Algérie, Algiers, Algeria; LPMM - Laboratoire de Parasitologie_Mycologie à la Faculté de Pharmacie, Monastir, Tunisia; BHU - Kala-azar Medical Research Centre, Banaras Hindu University, Varanasi, India. (DOC) [file pntd.0001155.s004.doc]

**Table S1: Designation and characteristics of *Leishmania* strains used in this study**

| **Lab-code** | **alt. code** | **origin** | **WHO-code** | **country** | **region** | **zymodeme^1^** | **clinical picture** | **Pop. *K*3 INF_NW+OW_^2^** | **Pop. *K*2 INF_NW_^3^** |  |
| --- | --- | --- | --- | --- | --- | --- | --- | --- | --- | --- |
| CHA-06 | ChaP1 | SENEPA | MCAN/PY/2000/L1P | Paraguay | Central Department | nd | CanL | 1 | 1 sub1B |  |
| CHA-08 | ChaP3 | SENEPA | MCAN/PY/2000/Meka | Paraguay | Central Department | nd | CanL | 1 | 1 sub1B |  |
| CHA-11 | ChaP6 | SENEPA | MCAN/PY/2000/PB2 | Paraguay | Central Department | nd | CanL | 1 | 1 sub1B |  |
| CHA-07 | ChaP2 | SENEPA | MCAN/PY/2000/L9P | Paraguay | Central Department | nd | CanL | 1 | 1 sub1A |  |
| CHA-09 | ChaP4 | SENEPA | MCAN/PY/2000/Doberman | Paraguay | Central Department | nd | CanL | 1 | 1 sub1A |  |
| CHA-10 | ChaP5 | SENEPA | MCAN/PY/2000/Dago | Paraguay | Central Department | nd | CanL | 1 | 1 sub1A |  |
| CHA-12 | ChaP7 | SENEPA | MCAN/PY/2000/Ringo | Paraguay | Central Department | nd | CanL | 1 | 1 sub1A |  |
| CHA-116 | IOCL 2987 | SENEPA | MHOM/PY/2006/AS1 | Paraguay | Asuncion | nd | VL | 1 | 1 sub1A |  |
| CHA-121 | IOCL 2992 | SENEPA | MHOM/PY/2007/AS6 | Paraguay | Asuncion | nd | VL | 1 | 1 sub1A |  |
| PG470 | 470 | SENEPA | MCAN/PY/2008/470 | Paraguay | Cordillera Department | nd | CanL | 1 | 1 sub1A |  |
| CHA-82 | IOCL 2688 | CLIOC | MCAN/BR/2002/JackCusteau | Brazil | Mato Grosso de Sul | IOC/Z1 | CanL | 1 | 1 sub1B |  |
| CHA-114 | IOCL 2985 | CLIOC | MHOM/BR/2007/JVF | Brazil | Mato Grosso do Sul | IOC/Z1 | VL | 1 | 1 sub1B |  |
| CHA-13 | M8787 | KIT | MCAN/BR/1984/CO910 | Brazil | Mato Grosso do Sul | MON-1 | CanL | 1 | 1 sub1B |  |
| CHA-75 | IOCL 2593 | CLIOC | MHOM/BR/2003/BSB | Brazil | Mato Grosso de Sul | IOC/Z1 | VL | 1 | 1 sub1B |  |
| CHA-115 | IOCL 2986 | CLIOC | MHOM/BR/2007/JFVL | Brazil | Mato Grosso do Sul | IOC/Z1 | VL | 1 | 1 sub1A |  |
| CHA-101 | IOCL 2665 | CLIOC | MCAN/BR/2002/LVV-136 | Brazil | Mato Grosso do Sul | IOC/Z1 | CanL | 1 | 1 sub1A |  |
| CHA-103 | IOCL 2672 | CLIOC | MCAN/BR/2002/LVV-147 | Brazil | Mato Grosso do Sul | IOC/Z1 | CanL | 1 | 1 sub1B |  |
| CHA-71 | IOCL 2579 | CLIOC | MHOM/BR/2003/WAZ | Brazil | Mato Grosso do Sul | IOC/Z1 | VL | 1 | 1 sub1B |  |
| CHA-83 | IOCL 2686 | CLIOC | MHOM/BR/2002/BGC | Brazil | Mato Grosso do Sul | IOC/Z1 | VL | 1 | 1 sub1B |  |
| CHA-67 | IOCL 2561 | CLIOC | MHOM/BR/2003/AAS | Brazil | Mato Grosso do Sul | IOC/Z1 | VL | 1 | 1 sub1B |  |
| CHA-68 | IOCL 2572 | CLIOC | MHOM/BR/2003/ALX | Brazil | Mato Grosso do Sul | IOC/Z1 | VL | 1 | 1 sub1B |  |
| CHA-69 | IOCL 2575 | CLIOC | MHOM/BR/2003/RMJ | Brazil | Mato Grosso do Sul | IOC/Z1 | VL | 1 | 1 sub1B |  |
| CHA-70 | IOCL 2576 | CLIOC | MHOM/BR/2003/DDG | Brazil | Mato Grosso do Sul | IOC/Z1 | VL | 1 | 1 sub1B |  |
| CHA-72 | IOCL 2581 | CLIOC | MHOM/BR/2003/JHS | Brazil | Mato Grosso do Sul | IOC/Z1 | VL | 1 | 1 sub1B |  |
| CHA-73 | IOCL 2584 | CLIOC | MHOM/BR/2003/JBIC | Brazil | Mato Grosso do Sul | IOC/Z1 | VL/HIV+ | 1 | 1 sub1B |  |
| CHA-74 | IOCL 2591 | CLIOC | MHOM/BR/2003/CAS | Brazil | Mato Grosso do Sul | IOC/Z1 | VL | 1 | 1 sub1B |  |
| CHA-76 | IOCL 2594 | CLIOC | MHOM/BR/2003/FCM | Brazil | Mato Grosso do Sul | IOC/Z1 | VL | 1 | 1 sub1B |  |
| CHA-79 | IOCL 2648 | CLIOC | MHOM/BR/2003/GJR | Brazil | Mato Grosso do Sul | IOC/Z1 | VL | 1 | 1 sub1B |  |
| CHA-81 | IOCL 2651 | CLIOC | MHOM/BR/2003/MAM | Brazil | Mato Grosso do Sul | IOC/Z1 | VL | 1 | 1 sub1B |  |
| CHA-84 | IOCL 2664 | CLIOC | MCAN/BR/2002/LVV-135 | Brazil | Mato Grosso do Sul | IOC/Z1 | CanL | 1 | 1 sub1B |  |
| CHA-80 | IOCL 2649 | CLIOC | MHOM/BR/2003/JT | Brazil | Mato Grosso do Sul | IOC/Z1 | VL | 1 | 1 sub1B |  |
| CHA-102 | IOCL 2668 | CLIOC | MCAN/BR/2002/LVV-139 | Brazil | Mato Grosso do Sul | IOC/Z1 | CanL | 1 | 1 sub1B |  |
| CHA-113 | IOCL 2984 | CLIOC | MHOM/BR/2007/LFSP | Brazil | Mato Grosso do Sul | IOC/Z1 | VL | 1 | 1 sub1B |  |
| CHA-137 | IOCL 3035 | CLIOC | MCAN/BR/2007/CG2 | Brazil | Mato Grosso do Sul | IOC/Z1 | CanL | 1 | 1 sub1B |  |
| CHA-47 | IOCL 2507 | CLIOC | MCAN/BR/2002/CP-TARZAN | Brazil | Espirito Santo | IOC/Z1 | CanL | 1 | 1 sub1A |  |
| CHA-51 | IOCL 957 | CLIOC | MHOM/BR/1987/HCO-1 | Brazil | Espírito Santo | IOC/Z1 | VL | 1 | 1 sub1A |  |
| CHA-53 | IOCL 956 | CLIOC | MHOM/BR/1987/HBG-2 | Brazil | Espírito Santo | IOC/Z1 | VL | 1 | 1 sub1A |  |
| CHA-55 | IOCL 963 | CLIOC | MHOM/BR/1987/HIT-1 | Brazil | Espírito Santo | IOC/Z1 | VL | 1 | 1 sub1A |  |
| CHA-94 | IOCL 2505 | CLIOC | MCAN/BR/2000/CNV-FEROZ | Brazil | Espírito Santo | IOC/Z1 | CanL | 1 | 1 sub1A |  |
| CHA-96 | IOCL 2508 | CLIOC | MCAN/BR/2003/CP-TAISON | Brazil | Espírito Santo | IOC/Z1 | CanL | 1 | 1 sub1A |  |
| CHA-98 | IOCL 2785 | CLIOC | MCAN/BR/2005/CP-40 | Brazil | Espírito Santo | IOC/Z1 | CanL | 1 | 1 sub1A |  |
| CHA-99 | IOCL 2789 | CLIOC | MHOM/BR/2005/HRNS-1 | Brazil | Espírito Santo | IOC/Z1 | VL | 1 | 1 sub1A |  |
| CHA-93 | IOCL 2504 | CLIOC | MHOM/BR/2001/HP-EMO | Brazil | Espírito Santo | IOC/Z1 | VL | 1 | 1 sub1A |  |
| CHA-95 | IOCL 2506 | CLIOC | MCAN/BR/2001/CP-SEMNOME | Brazil | Espírito Santo | IOC/Z1 | CanL | 1 | 1 sub1A |  |
| CHA-62 | IOCL 2647 | CLIOC | MHOM/BR/2003/ACS | Brazil | Pernambuco | IOC/Z1 | VL | 1 | 1 sub1A |  |
| CHA-104 | IOCL 2763 | CLIOC | MCAN/BR/2005/FACHIDE | Brazil | Pernambuco | IOC/Z1 | CanL | 1 | 1 sub1A |  |
| CHA-110 | IOCL 2933 | CLIOC | MCAN/BR/2006/MAIKE | Brazil | Pernambuco | IOC/Z1 | CanL | 1 | 1 sub1A |  |
| CHA-112 | IOCL 2983 | CLIOC | MCAN/BR/2007/SPOK II | Brazil | Pernambuco | IOC/Z1 | CanL | 1 | 1 sub1B |  |
| CHA-59 | IOCL 1848 | CLIOC | MCAN/BR/1991/1194BAJE | Brazil | Bahia | IOC/Z1 | CanL | 1 | 1 sub1A |  |
| CHA-01 |  | KIT | MHOM/BR/1974/PP75^T^ | Brazil | Bahia | MON-1 | VL | 1 | 1 sub1A |  |
| CHA-61 | IOCL 1849 | CLIOC | MCAN/BR/1991/1373BAJE | Brazil | Bahia | IOC/Z1 | CanL | 1 | 1 sub1A |  |
| CHA-43 | IOCL 45 | CLIOC | MCAN/BR/1980/CR3 | Brazil | Rio de Janeiro | IOC/Z1 | CanL | 1 | 1 sub1A |  |
| CHA-134 | IOCL 3015 | CLIOC | MHOM/BR/2007/WC | Brazil | Rio de Janeiro | IOC/Z1 | VL/HIV+ | 1 | 1 sub1A |  |
| CHA-138 | IOCL 3020 | CLIOC | MHOM/BR/2007/JFF_BM | Brazil | Rio de Janeiro | IOC/Z1 | VL/HIV+ | 1 | 1 sub1A |  |
| CHA-60 | IOCL 906 | CLIOC | MHOM/BR/1987/H-136 | Brazil | Ceará | IOC/Z1 | VL | 1 | 1 sub1A |  |
| CHA-52 | IOCL 329 | CLIOC | MCAN/BR/1984/17.206 | Brazil | Ceará | IOC/Z1 | CanL | 1 | 1 sub1A |  |
| CHA-54 | IOCL 667 | CLIOC | MHOM/BR/1986/H-33 | Brazil | Ceará | IOC/Z1 | VL | 1 | 1 sub1A |  |
| CHA-56 | IOCL 902 | CLIOC | MCAN/BR/1987/CCC18.406 | Brazil | Ceará | IOC/Z1 | CanL | 1 | 1 sub1A |  |
| CHA-64 | IOCL 665 | CLIOC | MCAN/BR/1986/CCC17.580 | Brazil | Ceará | IOC/Z1 | CanL | 1 | 1 sub1A |  |
| CHA-20 | M12734 | LSHTM | MHOM/BR/1989/M12734 | Brazil | Piauí Teresina | nd | VL | 1 | 1 sub1A |  |
| CHA-21 | M12737 | LSHTM | MCAN/BR/1989/M12737 | Brazil | Piauí Teresina | nd | CanL | 1 | 1 sub1A |  |
| CHA-19 | M12727 | LSHT | MCAN/BR/1989/M12727 | Brazil | Piauí Teresina | nd | CanL | 1 | 1 sub1A |  |
| CHA-63 | IOCL 2543 | CLIOC | MCAN/BR/2002/RN-CEPA2 | Brazil | Rio Grande do Norte | IOC/Z1 | CanL | 1 | 1 sub1A |  |
| CHA-57 | IOCL 2544 | CLIOC | MCAN/BR/2002/RN-CEPA3 | Brazil | Rio Grande do Norte | IOC/Z1 | CanL | 1 | 1 sub1A |  |
| CHA-03 | M9702 | KIT | MHOM/BR/1985/M9702 | Brazil | Pará Marajó | nd | VL | 1 | 1 sub1A |  |
| CHA-22 | M7633 | LSHTM | MCER/BR/1983/M7633 | Brazil | Pará Belém | nd | fox | 1 | 1 sub1B |  |
| CHA-23 | M12085 | LSHTM | MCER/BR/1989/M12085 | Brazil | Pará Belém | nd | fox | 1 | 1 sub1A/B |  |
| CHA-24 | M12084 | LSHTM | MCER/BR/1989/M12084 | Brazil | Pará Belém | nd | fox | 1 | 1 sub1A/B |  |
| CHA-25 | M6445 | LSHTM | MCER/BR/1981/M6445 | Brazil | Pará Belém | nd | fox | 1 | 1 sub1B |  |
| CHA-04 | LEM575 | JRCL | MHOM/BR/0000/Edmael | Brazil | nd | MON-1 | nd | 1 | 1 sub1B |  |
| CHA-14 | M8270 | KIT | MHOM/BR/1984/M8270 | Brazil | nd | nd | VL | 1 | 1 sub1A |  |
| CHA-15 | M1287 | KIT | MHOM/BR/1973/M1287 | Brazil | nd | nd | nd | 1 | 1 sub1A |  |
| CHA-27 | HN560 | LSHTM | MHOM/HN/1994/560 | Honduras | El Tigre | nd | CL | 1 | 1 sub1A |  |
| CHA-35 | HN419 | LSHTM | MHOM/HN/1994/419 | Honduras | San Juan Bautista | nd | CL | 1 | 1 sub1A |  |
| CHA-34 | HN412 | LSHTM | MHOM/HN/1994/412 | Honduras | nd | nd | CL | 1 | 1 sub1A |  |
| CHA-29 | HN122 | LSHTM | MHOM/HN/1988/122 | Honduras | El Tigre | nd | CL | 1 | 1 sub1A |  |
| CHA-30 | HN167 | LSHTM | MHOM/HN/1989/167 | Honduras | Orcuina | nd | CL | 1 | 1 sub1A |  |
| CHA-32 | HN336 | LSHTM | MHOM/HN/1993/336 | Honduras | San Juan Bautista | nd | CL | 1 | 1 sub1A |  |
| CHA-38 | HN504 | LSHTM | MHOM/HN/1994/504 | Honduras | El Tigre | nd | CL | 1 | 1 sub1A |  |
| CHA-42 | HN556 | LSHTM | MHOM/HN/1994/556 | Honduras | El Tigre | nd | CL | 1 | 1 sub1A |  |
| CHA-31 | HN310 | LSHTM | MHOM/HN/1993/310 | Honduras | San Juan Bautista | nd | CL | 1 | 1 sub1A |  |
| CHA-28 | HN115 | LSHTM | MHOM/HN/1988/115 | Honduras | El Tigre | nd | CL | 1 | 1 sub1A |  |
| CHA-36 | HN421 | LSHTM | MHOM/HN/1994/421 | Honduras | San Juan Bautista | nd | CL | 1 | 1 sub1A |  |
| CHA-37 | HN463 | LSHTM | MHOM/HN/1994/463 | Honduras | San Juan Bautista | nd | CL | 1 | 1 sub1A |  |
| CHA-41 | HN552 | LSHTM | MHOM/HN/1994/552 | Honduras | El Tigre | nd | CL | 1 | 1 sub1A |  |
| CHA-26 | HN29 | LSHTM | MHOM/HN/1987/29 | Honduras | San Francisco de Coray | nd | VL | 1 | 1 sub1A |  |
| CHA-33 | HN354 | LSHTM | MHOM/HN/1993/354 | Honduras | Alubaren | nd | VL | 3 | 2 sub2A |  |
| CHA-02 |  | KIT | MHOM/PA/1979/WR317 | Panama | nd | nd | CL | 3 | 2 sub2A |  |
| CHA-18 | WR285 | LSHTM | MHOM/PA/1978/W285 | Panama | nd | nd | CL | 3 | 2 sub2A |  |
| CHA-05 |  | JRCL | MHOM/CR/199**?**/LVCR | Costa Rica | Guanacaste | nd | nd | 3 | 2 sub2A |  |
| CHA-87 |  | CNRFV | MCAN/VE/2006/UCNA/LV2 | Venezuela | Aragua | nd | CanL | 3 | 2 sub2B |  |
| CHA-85 |  | CNRFV | MHOM/VE/2001/LV10 | Venezuela | Carabobo | nd | VL | 3 | 2 sub2B |  |
| CHA-86 |  | CNRFV | MHOM/VE/2004/IB-LAT | Venezuela | Guárico | nd | CL | 3 | 2 sub2B |  |
| CHA-89 |  | CNRFV | MHOM/VE/2000/LV04 | Venezuela | Carabobo | nd | VL | 3 | 2 sub2B |  |
| CHA-90 |  | CNRFV | MHOM/VE/2001/LV06 | Venezuela | Carabobo | nd | VL | 3 | 2 sub2B |  |
| CHA-65 | IOCL 1238 | CLIOC | MCAN/CO/1986/CL-223 | Colombia | Huila | IOC/Z1 | CanL | 1 | 1 sub1B |  |
| CHA-66 | IOCL 1236 | CLIOC | MHOM/CO/1984/CL-044 | Colombia | Cundinamarca | IOC/Z1 | VL | ‘1/3 | 1/2 sub1B |  |
| CHA-92 | IOCL 1243 | CLIOC | MDID/CO/1988/CL-490 | Colombia | Tolima | IOC/Z1 | *Didelphis* | 1 | 1 sub1B |  |
| INF-41 |  | CNRL | MHOM/ES/1993/PM1 | Spain | Majorca | MON-1 | VL/HIV+ | 1 | na |  |
| INF-43 |  | CNRL | MHOM/ES/1986/BCN16 | Spain | Catalonia | MON-1 | CL | 1 | na |  |
| ES1 (I) |  | ISCM | MHOM/ES/2001/LLM-984 | Spain | Madrid | nd | VL/transp. | 3 | na |  |
| ES2 (I) |  | ISCM | MHOM/ES/2001/LLM-983 | Spain | Madrid | MON-1 | VL/HIV+ | 1 | na |  |
| ES3 (I) |  | ISCM | MHOM/ES/2001/LLM-980 | Spain | Madrid | nd | VL | 3 | na |  |
| ES4 (I) |  | ISCM | MHOM/ES/2002/LLM-1181 | Spain | Madrid | MON-1 | VL/HIV+ | 1 | na |  |
| ES5 (I) |  | ISCM | MHOM/ES/2002/LLM-1212 | Spain | Madrid | MON-1 | VL/HIV+ | 1 | na |  |
| ES6 (I) |  | ISCM | MHOM/ES/2002/LLM-1166 | Spain | Madrid | nd | VL/HIV+ | 3 | na |  |
| ES7 (I) |  | ISCM | MCAN/ES/2001/LLM-1006 | Spain | Madrid | MON-1 | CanL | 1 | na |  |
| ES8 (I) |  | ISCM | MCAN/ES/2001/LLM-1014 | Spain | Madrid | MON-1 | CanL | 1 | na |  |
| ES9 (I) |  | ISCM | MCAN/ES/2001/LLM-1037 | Spain | Madrid | MON-1 | CanL | 1 | na |  |
| ES10 (I) |  | ISCM | MCAN/ES/2001/LLM-1040 | Spain | Madrid | MON-1 | CanL | 1 | na |  |
| ES11 (I) |  | ISCM | MHOM/ES/2001/LLM-981 | Spain | Majorca | MON-1 | VL/HIV+ | 1 | na |  |
| ES12 (I) |  | ISCM | MHOM/ES/2002/LLM-1122 | Spain | Majorca | MON-1 | VL/HIV+ | 1 | na |  |
| ES13 (I) |  | ISCM | MHOM/ES/2001/LLM-1048 | Spain | Majorca | MON-1 | VL/HIV+ | 1 | na |  |
| ES14 (I) |  | ISCM | MHOM/ES/2001/LLM-1049 | Spain | Majorca | MON-1 | VL/HIV+ | 1 | na |  |
| ES15 (I) |  | ISCM | MHOM/ES/2002/LLM-1150 | Spain | Majorca | MON-1 | VL/HIV+ | 1 | na |  |
| ES16 (I) |  | ISCM | MHOM/ES/2002/LLM-1109 | Spain | Majorca | MON-1 | VL | 1 | na |  |
| ES17 (I) |  | ISCM | MCAN/ES/2001/LLM-1008 | Spain | Majorca | MON-1 | CanL | 1 | na |  |
| ES18 (I) |  | ISCM | MCAN/ES/2001/LLM-1007 | Spain | Majorca | MON-1 | CanL | 1 | na |  |
| ES19 (I) |  | ISCM | MCAN/ES/2001/LLM-1038 | Spain | Majorca | MON-1 | CanL | 1 | na |  |
| ES20 (I) |  | ISCM | MCAN/ES/2002/LLM-1149 | Spain | Ibiza | MON-1 | CanL | 1 | na |  |
| ES21 (I) |  | ISCM | MCAN/ES/2002/LLM-1155 | Spain | Ibiza | MON-1 | CanL | 1 | na |  |
| ES22 (I) |  | ISCM | MCAN/ES/2002/LLM-1203 | Spain | Ibiza | MON-1 | CanL | 1 | na |  |
| ES23 (I) |  | ISCM | MCAN/ES/2002/LLM-1139 | Spain | Ibiza | MON-1 | CanL | 1 | na |  |
| ES24 (I) |  | ISCM | MCAN/ES/2002/LLM-1141 | Spain | Ibiza | MON-1 | CanL | 1 | na |  |
| ES25 (I) |  | ISCM | MCAN/ES/2002/LLM-1158 | Spain | Ibiza | MON-1 | CanL | 1 | na |  |
| ES 1 (II) |  | ISCM | MCAN/ES/2001/LLM-1068 | Spain | Madrid | MON-1 | CanL | 1 | na |  |
| ES 3 (II) |  | ISCM | MCAN/ES/2001/LLM-1106 | Spain | Madrid | MON-1 | CanL | 1 | na |  |
| ES 5 (II) |  | ISCM | MCAN/ES/2002/LLM-1113 | Spain | Madrid | MON-1 | CanL | 1 | na |  |
| ES 6 (II) |  | ISCM | MCAN/ES/2001/LLM-1116 | Spain | Madrid | MON-1 | CanL | 1 | na |  |
| ES 7 (II) |  | ISCM | MCAN/ES/2001/LLM-1128 | Spain | Madrid | MON-1 | CanL | 1 | na |  |
| ES 8 (II) |  | ISCM | MCAN/ES/2001/LLM-1136 | Spain | Madrid | MON-1 | CanL | 1 | na |  |
| ES 9 (II) |  | ISCM | MCAN/ES/2001/LLM-1148 | Spain | Madrid | MON-1 | CanL | 1 | na |  |
| ES 10 (II) |  | ISCM | MCAN/ES/2003/LLM-1228 | Spain | Ibiza | MON-1 | CanL | 1 | na |  |
| ES 11 (II) |  | ISCM | MCAN/ES/2003/LLM-1233 | Spain | Ibiza | MON-1 | CanL | 1 | na |  |
| ES 12 (II) |  | ISCM | MCAN/ES/2003/LLM-1238 | Spain | Ibiza | MON-1 | CanL | 1 | na |  |
| ES 13 (II) |  | ISCM | MCAN/ES/2003/LLM-1240 | Spain | Ibiza | MON-1 | CanL | 1 | na |  |
| ES 14 (II) |  | ISCM | MCAN/ES/2001/LLM-1215 | Spain | Ibiza | MON-1 | CanL | 1 | na |  |
| ES 15 (II) |  | ISCM | MCAN/ES/2003/LLM-1237 | Spain | Ibiza | MON-1 | CanL | 1 | na |  |
| ES 16 (II) |  | ISCM | MCAN/ES/2003/LLM-1241 | Spain | Ibiza | MON-1 | CanL | 1 | na |  |
| ES 17 (II) |  | ISCM | MCAN/ES/2003/LLM-1226 | Spain | Ibiza | MON-1 | CanL | 1 | na |  |
| ES 18 (II) |  | ISCM | MCAN/ES/2003/LLM-1267 | Spain | Ibiza | MON-1 | CanL | 1 | na |  |
| ES 1 (III) |  | ISCM | MHOM/ES/2002/LLM-1220 | Spain | Madrid | MON-1 | VL/HIV+ | 1 | na |  |
| ES 2 (III) |  | ISCM | MHOM/ES/2002/LLM-1217 | Spain | Madrid | MON-1 | VL/HIV+ | 1 | na |  |
| ES 3 (III) |  | ISCM | MHOM/ES/2001/LLM-1036 | Spain | Madrid | MON-27 | VL/HIV+ | 3 | na |  |
| ES 4 (III) |  | ISCM | MHOM/ES/2003/LLM-1254 | Spain | Madrid | nd | VL/HIV+ | 3 | na |  |
| ES 5 (III) |  | ISCM | MHOM/ES/1997/LLM-707 | Spain | Madrid | MON-24 | VL/HIV+ | 3 | na |  |
| ES 6 (III) |  | ISCM | MHOM/ES/1997/LLM-709 | Spain | Madrid | MON-24 | VL/HIV+ | 3 | na |  |
| ES 7 (III) |  | ISCM | MHOM/ES/2002/LLM-1184 | Spain | Barcelona | MON-1 | VL | 1 | na |  |
| ES 8 (III) |  | ISCM | MHOM/ES/2003/LLM-1232 | Spain | Barcelona | MON-1 | VL/HIV+ | 1 | na |  |
| ES 9 (III) |  | ISCM | MHOM/ES/1998/LLM-810 | Spain | Majorca | MON-24 | VL/HIV+ | 3 | na |  |
| ES10 (III) |  | ISCM | MHOM/ES/1999/LLM-846 | Spain | Majorca | MON-24 | VL/HIV+ | 3 | na |  |
| ES11 (III) |  | ISCM | MHOM/ES/1998/LLM-745 | Spain | Andalusia | MON-34 | VL/HIV+ | 3 | na |  |
| ES12 (III) |  | ISCM | MHOM/ES/1999/LLM-879 | Spain | Andalusia | MN-34 | VL/HIV+ | 3 | na |  |
| ES13 (III) |  | ISCM | MHOM/ES/2001/LLM-1035 | Spain | Majorca | MON-1 | VL/HIV+ | 1 | na |  |
| ES14 (III) |  | ISCM | MHOM/ES/2002/LLM-1167 | Spain | Majorca | MON-1 | VL/HIV+ | 1 | na |  |
| ES15 (III) |  | ISCM | MHOM/ES/1995/LLM-531 | Spain | Madrid | MON-34 | VL/HIV+ | 3 | na |  |
| ES16 (III) |  | ISCM | MHOM/ES/1998/LLM-780 | Spain | Madrid | MON-34 | VL/HIV+ | 3 | na |  |
| ES17 (III) |  | ISCM | MHOM/ES/2001/LLM-1034 | Spain | Madrid | MON-34 | VL/HIV+ | 3 | na |  |
| INF-32 |  | CNRLM | MCAN/ES/1986/LEM935 | Spain | Poboleda | MON-77 | CanL | 1 | na |  |
| INF-04 |  | KIT | MHOM/ES/1987/Lombardi | Spain | nd | MON-24 | CL | 3 | na |  |
| INF-37 | LG 19 | CNRLM | MHOM/ES/1988/LLM175 | Spain | Madrid | MON-198 | VL/HIV+ | 3 | na |  |
| INF-46 | LG 8 | LSHTM | MHOM/ES/1991/LEM2298 | Spain | Valencia | MON-183 | VL/HIV+ | 3 | na |  |
| INF-55 | LG 20 | CNRLM | MHOM/ES/1992/LLM373 | Spain | Madrid | MON-199 | VL/HIV+ | 3 | na |  |
| PT1 (I) |  | IHMT | MCAN/PT/1993/IMT193 | Portugal | Algarve | MON-1 | CanL | 1 | na |  |
| PT2 (I) |  | IHMT | IPERN/PT/1993/IMT189 | Portugal | Algarve | MON-1 | sandfly | 1 | na |  |
| PT3 (I) |  | IHMT | MCAN/PT/1995/IMT205 | Portugal | Alentejo | MON-1 | CanL | 1 | na |  |
| PT4 (I) |  | IHMT | MCAN/PT/2003/IMT328 | Portugal | Alentejo | MON-1 | CanL | 1 | na |  |
| PT5 (I) |  | IHMT | MCAN/PT/89/IMT162 | Portugal | Alto Douro | MON-1 | CanL | 1 | na |  |
| PT6 (I) |  | IHMT | IARI/PT/1989/IMT169 | Portugal | Alto Douro | MON-1 | sandfly | 1 | na |  |
| PT7 (I) |  | IHMT | IARI/PT/1989/IMT170 | Portugal | Alto Douro | MON-1 | sandfly | 1 | na |  |
| PT8 (I) |  | IHMT | MHOM/PT/2002/IMT279 | Portugal | Alto Douro | MON-1 | VL | 1 | na |  |
| PT9 (I) |  | IHMT | MHOM/PT/2002/IMT288 | Portugal | Alto Douro | MON-1 | VL | 1 | na |  |
| PT10 (I) |  | IHMT | MHOM/PT/2003/IMT337 | Portugal | Alto Douro | MON-1 | CL | 1 | na |  |
| PT11 (I) |  | IHMT | MCAN/PT/1997/IMT229 | Portugal | Lisbon | MON-1 | CanL | 1 | na |  |
| PT12 (I) |  | IHMT | MCAN/PT/2003/IMT300 | Portugal | Lisbon | MON-1 | CanL | 1 | na |  |
| PT13 (I) |  | IHMT | MCAN/PT/2003/IMT327 | Portugal | Lisbon | MON-1 | CanL | 1 | na |  |
| PT14 (I) |  | IHMT | MCAN/PT/2003/IMT316 | Portugal | Lisbon | MON-1 | CanL | 1 | na |  |
| PT15 (I) |  | IHMT | MCAN/PT/2003/IMT329 | Portugal | Lisbon | MON-1 | CanL | 1 | na |  |
| PT16 (I) |  | IHMT | MCAN/PT/2003/IMT330 | Portugal | Lisbon | MON-1 | CanL | 1 | na |  |
| PT17 (I) |  | IHMT | MCAN/PT/2003/IMT338 | Portugal | Lisbon | MON-1 | CanL | 1 | na |  |
| PT18 (I) |  | IHMT | MCAN/PT/2003/IMT339 | Portugal | Lisbon | MON-1 | CanL | 1 | na |  |
| PT19 (I) |  | IHMT | MHOM/PT/1989/IMT163 | Portugal | Lisbon | MON-1 | VL/HIV+ | 1 | na |  |
| PT20 (I) |  | IHMT | MHOM/PT/2002/IMT293 | Portugal | Lisbon | MON-1 | VL/HIV+ | 1 | na |  |
| PT21 (I) |  | IHMT | MHOM/PT/2003/IMT293-B | Portugal | Lisbon | MON-1 | VL/HIV+ | 1 | na |  |
| PT22 (I) |  | IHMT | MHOM/PT/2002/IMT294 | Portugal | Lisbon | MON-1 | VCL/HIV+ | 1 | na |  |
| PT23 (I) |  | IHMT | MHOM/PT/ 2002/IMT296 | Portugal | Lisbon | MON-1 | VL/HIV+ | 1 | na |  |
| PT24 (I) |  | IHMT | MHOM/PT/2003/IMT299 | Portugal | Lisbon | MON-1 | VL/HIV+ | 1 | na |  |
| PT25 (I) |  | IHMT | MHOM/PT/1998/IMT238 | Portugal | Lisbon | MON-80 | VL/HIV+ | 3 | na |  |
| PT 1 (II) |  | IHMT | MHOM/PT/2000/IMT262 | Portugal | Lisbon | MON-1 | VL/HIV+ | 1 | na |  |
| PT 2 (II) |  | IHMT | MHOM/PT/2000/IMT262-A | Portugal | Lisbon | MON-1 | VL/HIV+ | 1 | na |  |
| PT 3 (II) |  | IHMT | MHOM/PT/1993/IMT184 | Portugal | Lisbon | MON-1 | VL/HIV+ | 1 | na |  |
| PT 4 (II) |  | IHMT | MHOM/PT/1988/IMT151 | Portugal | Lisbon | MON-1 | VL | 1 | na |  |
| PT 5 (II) |  | IHMT | MHOM/PT/2004/IMT362 | Portugal | Lisbon | MON-1 | CL/HIV+ | 1 | na |  |
| PT 6 (II) |  | IHMT | MHOM/PT/2004/IMT364 | Portugal | Lisbon | MON-1 | VL/HIV+ | 1 | na |  |
| PT 7 (II) |  | IHMT | MHOM/PT/2004/IMT359 | Portugal | Alto Douro | MON-1 | VL | 1 | na |  |
| PT 8 (II) |  | IHMT | MHOM/PT/2004/IMT360 | Portugal | Alto Douro | MON-1 | VL | 1 | na |  |
| PT 9 (II) |  | IHMT | MHOM/PT/2004/IMT363 | Portugal | Alentejo | nd | VL | 3 | na |  |
| PT 10 (II) |  | IHMT | MCAN/PT/2003/IMT329 | Portugal | Lisbon | MON-1 | CanL | 1 | na |  |
| PT 11 (II) |  | IHMT | MCAN/PT/2003/IMT331 | Portugal | Lisbon | MON-1 | CanL | 1 | na |  |
| PT 12 (II) |  | IHMT | MCAN/PT/2003/IMT354 | Portugal | Lisbon | MON-1 | CanL | 1 | na |  |
| PT 13 (II) |  | IHMT | MCAN/PT/1989/IMT160 | Portugal | Alto Douro | MON-1 | CanL | 1 | na |  |
| PT 14 (II) |  | IHMT | MCAN/PT/1989/IMT161 | Portugal | Alto Douro | MON-1 | CanL | 1 | na |  |
| PT 15 (II) |  | IHMT | MCAN/PT/2004/IMT355 | Portugal | Alentejo | MON-1 | CanL | 1 | na |  |
| PT 16 (II) |  | IHMT | MCAN/PT/2004/IMT356 | Portugal | Alentejo | MON-1 | CanL | 1 | na |  |
| PT 17 (II) |  | IHMT | MCAN/PT/1994/IMT204 | Portugal | Algarve | MON-1 | CanL | 1 | na |  |
| PT 18 (II) |  | IHMT | MVUL/PT/1982/IMT108 | Portugal | Lisbon | MON-1 | fox | 1 | na |  |
| INF-44 | LG 6 | CNRLM | MHOM/PT/2000/IMT260 | Portugal | Lisbon | MON-1 | CL | 1 | na |  |
| INF-39 | LG 1 | CNRLM | MHOM/FR/1978/LEM75 | France | Languedoc | MON-1 | VL | 1 | na |  |
| INF-40 | LG 2 | CNRLM | MHOM/FR/1995/LPN114 | France | Côte d'Azur | MON-1 | VL | 2 | na |  |
| INF-42 | LG 4 | CNRLM | MHOM/FR/1997/LSL29 | France | Languedoc | MON-1 | CL | 1 | na |  |
| INF-35 |  | CNRLM | MCAN/FR/1987/RM1 | France | Marseille | MON-108 | CanL | 1 | na |  |
| INF-03 |  | KIT | MHOM/FR/1962/LRC-L47 | France | nd | nd | VL | 3 | na |  |
| INF-45 | LG 7 | CNRLM | MHOM/FR/1996/LEM3249 | France | Roussillion | MON-29 | CL | 3 | na |  |
| INF-47 | LG 14 | CNRLM | MHOM/FR/1980/LEM189 | France | Roussillion | MON-11 | CL | 3 | na |  |
| INF-77 |  | CNRLM | MHOM/FR/1985/LPN24 | France | Corse | MON-1 | CL | 2 | na |  |
| INF-78 |  | CNRLM | MHOM/FR/1987/LEM1163-CL | France | Cévennes | MON-1 | VL | 1 | na |  |
| INF-79 |  | CNRLM | MHOM/FR/1987/LEM1224 | France | Cévennes | MON-1 | VL | 1 | na |  |
| INF-80 |  | CNRLM | MHOM/FR/1988/LEM1345 | France | Cévennes | MON-1 | VL | 1 | na |  |
| INF-100 |  | CNRLM | MHOM/FR/1988/CRE2 | France | Corse | MON-1 | VL | 2 | na |  |
| INF-81 |  | CNRLM | MHOM/FR/1989/LEM1614 | France | Cévennes | MON-1 | VL | 1 | na |  |
| INF-82 |  | CNRLM | MHOM/FR/1989/LPN58 | France | Côte d’Azur | MON-1 | VL | 1 | na |  |
| INF-83 |  | CNRLM | MCAN/FR/1989/LPN57 | France | Côte d’Azur | MON-1 | CanL | 2 | na |  |
| INF-84 |  | CNRLM | MHOM/FR/1990/LPN61 | France | Côte d’Azur | MON-1 | VL | 2 | na |  |
| INF-85 |  | CNRLM | MHOM/FR/1990/LPN62 | France | Côte d’Azur | MON-1 | VL | 2 | na |  |
| INF-86 |  | CNRLM | MHOM/FR/1990/LPN66 | France | Côte d’Azur | MON-1 | VL | 2 | na |  |
| INF-87 |  | CNRLM | MHOM/FR/1990/LEM2191 | France | Cévennes | MON-1 | VL | 1 | na |  |
| INF-88 |  | CNRLM | MHOM/FR/1991/LEM2327 | France | Pyrénées-Orientales | MON-1 | VL | 1 | na |  |
| INF-89 |  | CNRLM | MHOM/FR/1993/LEM2652 | France | Pyrénées-Orientales | MON-1 | VL | 1 | na |  |
| INF-90 |  | CNRLM | MHOM/FR1994/LEM2859 | France | Pyrénées-Orientales | MON-1 | CL | 1 | na |  |
| INF-91 |  | CNRLM | MHOM/FR/1995/LEM2982 | France | Pyrénées-Orientales | MON-1 | CL | 1 | na |  |
| INF-92 |  | CNRLM | MHOM/FR/1995/LEM3003 | France | Pyrénées-Orientales | MON-1 | CL | 1 | na |  |
| INF-93 |  | CNRLM | MHOM/FR/1996/LPM138 | France | Provence | MON-1 | VL/HIV+ | 1 | na |  |
| INF-94 |  | CNRLM | MHOM/FR/1996/LPM154 | France | Provence | MON-1 | VL | 1 | na |  |
| INF-95 |  | CNRLM | MHOM/FR/1996/LEM3276 | France | Provence | MON-1 | VL | 1 | na |  |
| INF-101 |  | CNRLM | MHOM/FR/1996/LPM161 | France | Provence | MON-1 | VL/HIV+ | 1 | na |  |
| INF-96 |  | CNRLM | MHOM/FR/1996/LEM3285 | France | Provence | MON-1 | VL | 1 | na |  |
| INF-97 |  | CNRLM | MHOM/FR/1997/LPN154 | France | Corsica | MON-1 | VL | 2 | na |  |
| INF-98 |  | CNRLM | MHOM/FR/1999/CRE103 | France | Corsica | MON-1 | VL | 1 | na |  |
| INF-99 |  | CNRLM | MHOM/FR/1999/LPM190 | France | Corsica | MON-1 | VL | 2 | na |  |
| INF-48 | LG 15 | CNRLM | MHOM/MT/1985/BUCK | Malta | Malta | MON-78 | CL | 3 | na |  |
| INF-56 | LG 21 | CNRLM | MHOM/IT/1994/ISS1036 | Italy | nd | MON-228 | VL | 3 | na |  |
| INF-57 | LG 22 | CNRLM | MHOM/IT/1993/ISS800 | Italy | Sicily | MON-188 | VL/HIV+ | 3 | na |  |
| INF-167 |  | ISS | IPRF/IT/1985/ISS174 | Italy | Abruzzo | MON-1 | sandfly | 1 | na |  |
| INF-168 |  | ISS | IPER/IT/1986/ISS231 | Italy | Puglia | MON-1 | sandfly | 1 | na |  |
| INF-169 |  | ISS | MHOM/IT/1998/ISS1779 | Italy | Puglia | MON-1 | VL/HIV+ | 1 | na |  |
| INF-170 |  | ISS | MHOM/IT/2006/ISS2826 | Italy | Puglia | MON-1 | VL/HIV+ | 1 | na |  |
| INF-171 |  | ISS | MCAN/IT/1993/ISS949 | Italy | Sicily | MON-1 | CanL | 1 | na |  |
| INF-172 |  | ISS | MHOM/IT/1996/ISS1435 | Italy | Sicily | MON-1 | CL | 2 | na |  |
| INF-173 |  | ISS | MHOM/IT/2002/ISS2179 | Italy | Sicily | MON-1 | VL/HIV+ | 1 | na |  |
| INF-174 |  | ISS | MCAN/IT/2002/ISS2420 | Italy | Sicily | MON-1 | CanL | 1 | na |  |
| INF-175 |  | ISS | MHOM/IT/2004/ISS2653 | Italy | Sicily | MON-1 | CL | 1 | na |  |
| INF-176 |  | ISS | IPER/IT/2005/2805 | Italy | Sicily | MON-1 | sandfly | 2 | na |  |
| INF-177 |  | ISS | MFEL/IT/2005/ISS2814 | Italy | Sicily | MON-1 | Feline L | 2 | na |  |
| INF-178 |  | ISS | MHOM/IT/1995/ISS1268 | Italy | Sardegna | MON-1 | VL/ HIV+ | 1 | na |  |
| INF-179 |  | ISS | MCAN/IT/1996/ISS1457 | Italy | Sardegna | MON-1 | CanL | 1 | na |  |
| INF-180 |  | ISS | MCAN/IT/2002/ISS2379 | Italy | Lazio | MON-1 | CanL | 1 | na |  |
| INF-181 |  | ISS | MHOM/IT/2002/ISS2434 | Italy | Lazio | MON-1 | VL/ HIV+ | 1 | na |  |
| INF-182 |  | ISS | MHOM/IT/2002/ISS2384 | Italy | Campania | MON-1 | VL | 1 | na |  |
| INF-183 |  | ISS | MHOM/IT/2002/ISS2426 | Italy | Campania | MON-1 | VL | 1 | na |  |
| INF-184 |  | ISS | MHOM/IT/2002/ISS2429 | Italy | Campania | MON-1 | CanL | 1 | na |  |
| INF-185 |  | ISS | MCAN/IT/2003/ISS2609 | Italy | Campania | MON-1 | CanL | 1 | na |  |
| INF-186 |  | ISS | MHOM/IT/2003/ISS2615 | Italy | Campania | MON-1 | VL | 2 | na |  |
| INF-187 |  | ISS | MHOM/IT/2003/ISS2641 | Italy | Campania | MON-1 | VL | 1 | na |  |
| INF-188 |  | ISS | MHOM/IT/2005/ISS 2786 | Italy | Campania | MON-1 | VL | 1 | na |  |
| INF-189 |  | ISS | MCAN/IT/2002/ISS2427 | Italy | Calabria | MON-1 | CanL | 1 | na |  |
| INF-190 |  | ISS | MHOM/IT/2002/ISS2452 | Italy | Molise | MON-1 | VL | 1 | na |  |
| INF-191 |  | ISS | MCAN/IT/2003/ISS2611 | Italy | Piemonte | MON-1 | CanL | 1 | na |  |
| INF-192 |  | ISS | MHOM/IT/2002/ISS2508 | Italy | Piemonte/ Lombardia | MON-1 | VL/HIV+ | 1 | na |  |
| INF-193 |  | ISS | MCAN/IT/2004/ISS2658 | Italy | Lombardia | MON-1 | CanL | 1 | na |  |
| INF-194 |  | ISS | MHOM/IT/2002/ISS2524 | Italy | Liguria | MON-1 | VL | 1 | na |  |
| GR1 |  | HPI | MHOM/GR/2001/GH1 | Greece | Athens | MON-1 | VL | 2 | na |  |
| GR2 |  | HPI | MHOM/GR/2001/GH2 | Greece | Athens | MON-1 | VL | 2 | na |  |
| GR3 |  | HPI | MHOM/GR/2001/GH3 | Greece | Crete | MON-1 | VL | 2 | na |  |
| GR4 |  | HPI | MHOM/GR/2001/GH5 | Greece | Crete | MON-1 | VL | 2 | na |  |
| GR5 |  | HPI | MHOM/GR/2001/GH6 | Greece | Athens | MON-98 | VL | 2 | na |  |
| GR7 |  | HPI | MHOM/GR/2001/GH8 | Greece | Athens | MON-1 | VL | 2 | na |  |
| GR8 |  | HPI | MHOM/GR/2001/GH9 | Greece | Athens | MON-1 | VL | 2 | na |  |
| GR9 |  | HPI | MHOM/GR/2001/GH10 | Greece | Athens | MON-1 | VCL/HIV+ | 2 | na |  |
| GR10 |  | HPI | MHOM/GR/2001/GH11 | Greece | Athens | MON-1 | VL | 2 | na |  |
| GR11 |  | HPI | MCAN/GR/2001/GD3 | Greece | Crete | MON-98 | CanL | 2 | na |  |
| GR12 |  | HPI | MCAN/GR/2001/GD4 | Greece | Crete | MON-98 | CanL | 2 | na |  |
| GR13 |  | HPI | MCAN/GR/2003/GD5 | Greece | Crete | MON-1 | CanL | 2 | na |  |
| GR14 |  | HPI | MCAN/GR/2001/GD7 | Greece | Crete | MON-1 | CanL | 2 | na |  |
| GR15 |  | HPI | MCAN/GR/2001/GD8 | Greece | Crete | MON-98 | CanL | 2 | na |  |
| GR16 |  | HPI | MHOM/GR/2002/GH12 | Greece | Crete | MON-1 | VL | 2 | na |  |
| INF-10 |  | JRCL | MCAN/TR/1996/EP16 | Turkey | nd | nd | CanL | 2 | na |  |
| INF-11 |  | JRCL | MHOM/TR/1994/EP3 | Turkey | nd | nd | nd | 2 | na |  |
| INF-12 |  | JRCL | MCAN/IL/1994/LRC-L639 | Israel | cIL / Nataf | MON-1 | CanL | 2 | na |  |
| INF-13 |  | JRCL | MCAN/IL/1996/LRC-L685 | Israel | cIL / Nataf | nd | CanL | 2 | na |  |
| INF-14 |  | JRCL | MCAN/IL/1996/LRC-L695 | Israel | cIL / Nili | nd | CanL | 2 | na |  |
| INF-15 |  | JRCL | MCAN/IL/1996/LRC-L709 | Israel | nIL/ Klil | nd | CanL | 2 | na |  |
| INF-16 |  | JRCL | MCAN/IL/1997/LRC-L716 | Israel | cIL / Nataf | nd | CanL | 2 | na |  |
| INF-17 |  | JRCL | MCAN/IL/1997/LRC-L717 | Israel | cIL / Nataf | nd | CanL | 2 | na |  |
| INF-18 |  | JRCL | MCAN/IL/1997/LRC-L718 | Israel | cIL / Nataf | nd | CanL | 2 | na |  |
| INF-20 |  | JRCL | MCAN/IL/1997/LRC-L719 | Israel | cIL / Rishon Lezion | nd | CanL | 2 | na |  |
| INF-21 |  | JRCL | MCAN/IL/1997/LRC-L720 | Israel | cIL / Tzur Natan | nd | CanL | 2 | na |  |
| INF-22 |  | JRCL | MCAN/IL/1996/LRC-L689 | Israel | cIL / Sataf | nd | CanL | 2 | na |  |
| INF-23 |  | JRCL | MCAN/IL/1996/LRC-L699 | Israel | nIL / Klil | nd | CanL | 2 | na |  |
| INF-24 |  | JRCL | MCAN/IL/1996/LRC-L700 | Israel | cIL / Nataf | nd | CanL | 2 | na |  |
| INF-25 |  | JRCL | MCAN/IL/1996/LRC-L705 | Israel | nIL / Klil | nd | CanL | 2 | na |  |
| INF-26 |  | JRCL | MCAN/IL/1996/LRC-L706 | Israel | nIL / Klil | nd | CanL | 2 | na |  |
| INF-27 |  | JRCL | MCAN/IL/1996/LRC-L708 | Israel | nIL / Klil | nd | CanL | 2 | na |  |
| INF-28 |  | JRCL | MHOM/PS/1999/LRC-L773 | Palestine | Jenin | MON-281 | VL | 2 | na |  |
| INF-30 |  | JRCL | MCAN/IL/1996/LRC-L692 | Israel | cIL / Nili | nd | CanL | 2 | na |  |
| INF-68 |  | JRCL | MCAN/IL/1991/LRC-L613 | Israel | nIL / Avtalyon | nd | CanL | 2 | na |  |
| INF-69 |  | JRCL | MCAN/IL/2005/LRC-L1252 | Israel | cIL / Na'ale | nd | CanL | 2 | na |  |
| INF-70 |  | JRCL | MCAN/IL/2005/LRC-L1253 | Israel | cIL / Bet Shemeh | nd | CanL | 2 | na |  |
| INF-71 |  | JRCL | MCAN/IL/2005/LRC-L1257 | Israel | cIL / Bet Nehemia | nd | CanL | 2 | na |  |
| INF-72 |  | JRCL | MCAN/IL/2005/LRC-L1275 | Israel | cIL/ Ben Shemen | nd | CanL | 2 | na |  |
| INF-73 |  | JRCL | MCAN/IL/2006/LRC-L1278 | Israel | cIL / Yehud | nd | CanL | 2 | na |  |
| INF-74 |  | JRCL | MCAN/IL/2005/LRC-L1280 | Israel | nIL / Kedarim | nd | CanL | 2 | na |  |
| INF-76 |  | JRCL | MCAN/IL/1997/Lychee8849 | Israel | nd | nd | CanL | 2 | na |  |
| INF-127 |  | JRCL | MCAN/IL/1996/LRC-L693 | Israel | cIL / Nili | nd | CanL | 2 | na |  |
| INF-128 |  | JRCL | MHOM/IL/1998/LRC-L742 | Israel | cIL / Barkan | nd | VL | 2 | na |  |
| INF-129 |  | JRCL | MCAN/IL/1998/LRC-L741 | Israel | cIL / Alei Zahav | nd | CanL | 2 | na |  |
| PS-SLD7 |  | AQNHI | MHOM/PS/2000/SL7 | Palestine | Hebron | nd | VL | 2 | na |  |
| PS-SLD8 |  | AQNHI | MHOM/PS/2000/SL8 | Palestine | Hebron | nd | VL | 2 | na |  |
| PS-SLD18 |  | AQNHI | MHOM/PS/2000/SL18 | Palestine | Hebron | nd | VL | 2 | na |  |
| PS-SLD20 |  | AQNHI | MHOM/PS/1993/SL20 | Palestine | Hebron | nd | VL | 2 | na |  |
| PS-SLD21 |  | AQNHI | MHOM/PS/2004/SL21 | Palestine | Hebron/Betola | nd | VL | 2 | na |  |
| PS-SLD22 |  | AQNHI | MHOM/PS/2005/SL22 | Palestine | Hebron/Noba | nd | VL | 2 | na |  |
| PS-SLD23 |  | AQNHI | MHOM/PS/2005/SL23 | Palestine | Hebron/Dersamet | nd | VL | 2 | na |  |
| PS-SLD26 |  | AQNHI | MHOM/PS/2006/SL26 | Palestine | Hebron | nd | VL | 2 | na |  |
| PS-SLD27 |  | AQNHI | MHOM/PS/2006/LRC-L1296 | Palestine | Hebron/Samoa | MON-1 | VL | 2 | na |  |
| LEM417 |  | IPA | MHOM/DZ/1982/LIPA59 | Algeria | nd | MON-24 | CL | 3 | na |  |
| Alg9 |  | IPA | MCAN/DZ/1998/LIPA882 | Algeria | Tizi Ouzou | MON-1 | CanL | 3 | na |  |
| Alg18 |  | IPA | MHOM/DZ/2000/LIPA1086 | Algeria | Boumerdes | MON-24 | CL | 3 | na |  |
| Alg48 |  | IPA | MHOM/DZ/1999/LIPA1066 | Algeria | Alger | MON-24 | CL | 3 | na |  |
| Alg45 |  | IPA | MHOM/DZ/1998/LIPA881 | Algeria | Tizi Ouzou | MON-24 | VL | 3 | na |  |
| Alg38 |  | IPA | MHOM/DZ/1995/LIPA459 | Algeria | Lakhdaria | MON-24 | CL | 3 | na |  |
| Alg16 |  | IPA | MHOM/DZ/1999/LIPA1058 | Algeria | Lakhdaria | MON-80 | CL | 3 | na |  |
| Alg22 |  | IPA | MCAN /DZ/2000/LIPA1118 | Algeria | Alger | MON-1 | CanL | 3 | na |  |
| Alg32 |  | IPA | MHOM/DZ/2002/LIPA1323 | Algeria | Tizi Ouzou | MON-1 | VL | 3 | na |  |
| Alg51 |  | IPA | MHOM/DZ/2001/LIPA1140 | Algeria | Boumerdes | MON-24 | CL | 3 | na |  |
| Alg62 |  | IPA | MHOM/DZ/2000/LIPA1087 | Algeria | Béjaia | MON-1 | VL | 3 | na |  |
| Alg39 |  | IPA | MHOM/DZ/1996/LIPA477 | Algeria | Ain Defla | MON-24 | CL | 3 | na |  |
| Alg29 |  | IPA | MHOM/DZ/2001/LIPA1226 | Algeria | Alger | MON-24 | CL | 3 | na |  |
| Alg12 |  | IPA | MHOM/DZ/1999/LIPA977 | Algeria | Tizi Ouzou | MON-24 | CL | 3 | na |  |
| Alg19 |  | IPA | MHOM/DZ/1995/LIPA 440 | Algeria | Alger | MON-24 | CL | 3 | na |  |
| Alg20 |  | IPA | MHOM/DZ/1995/LIPA452 | Algeria | Boumerdes | MON-24 | CL | 3 | na |  |
| Alg34 |  | IPA | MHOM/DZ/2002/LIPA1338 | Algeria | Lakhdaria | MON-1 | VL | 2 | na |  |
| Alg54 |  | IPA | MHOM/DZ/2001/LIPA1233 | Algeria | Béjaia | MON-1 | VL | 2 | na |  |
| Alg30 |  | IPA | MHOM/DZ/2001/LIPA1227 | Algeria | Bejaia | MON-1 | VL | 2 | na |  |
| Alg24 |  | IPA | MHOM/DZ/2001/LIPA1148 | Algeria | Bouira | MON-1 | VL | 2 | na |  |
| Alg26 |  | IPA | MCAN/DZ/2001/LIPA1179 | Algeria | Alger | MON-1 | CanL | 2 | na |  |
| Alg28 |  | IPA | MCAN/DZ/2001/LIPA1213 | Algeria | Alger | MON-1 | CanL | 2 | na |  |
| Alg49 |  | IPA | MCAN/DZ/2000/LIPA1109 | Algeria | Alger | MON-1 | CanL | 2 | na |  |
| Alg6 |  | IPA | MHOM/DZ/1998/LIPA842 | Algeria | Ain-Defla | MON-1 | VL | 2 | na |  |
| Alg7 |  | IPA | MHOM/DZ/1998/LIPA851 | Algeria | Blida | MON-1 | VL | 2 | na |  |
| Alg8 |  | IPA | MHOM/DZ/1998/LIPA867 | Algeria | Alger | MON-1 | VL | 2 | na |  |
| Alg10 |  | IPA | MCAN/DZ/1998/LIPA904 | Algeria | Alger | MON-1 | CanL | 2 | na |  |
| Alg11 |  | IPA | MCAN/DZ/1998/LIPA911 | Algeria | Alger | MON-1 | CanL | 2 | na |  |
| Alg13 |  | IPA | MHOM/DZ/1999/LIPA979 | Algeria | Tizi Ouzou | MON-1 | VL | 2 | na |  |
| Alg15 |  | IPA | MHOM/DZ/1999/LIPA1002 | Algeria | Tizi Ouzou | MON-1 | VL | 2 | na |  |
| Alg21 |  | IPA | MCAN/DZ/2000/LIPA1113 | Algeria | Alger | MON-1 | CanL | 2 | na |  |
| Alg36 |  | IPA | MHOM/DZ/2002/LIPA1342 | Algeria | Blida | MON-1 | VL | 2 | na |  |
| Alg50 |  | IPA | MCAN/DZ/2000/LIPA1117 | Algeria | Alger | MON-1 | CanL | 2 | na |  |
| Alg60 |  | IPA | MCAN/DZ/2002/LIPA1341 | Algeria | Alger | MON-1 | CanL | 2 | na |  |
| Alg61 |  | IPA | MHOM/DZ/2002/LIPA1343 | Algeria | Blida | MON-1 | VL | 2 | na |  |
| Alg37 |  | IPA | MHOM/DZ/1995/LIPA448 | Algeria | Setif | MON-1 | VL | 2 | na |  |
| Alg44 |  | IPA | MHOM/DZ/1996/LIPA529 | Algeria | Tizi Ouzou | MON-1 | VL | 2 | na |  |
| Alg40 |  | IPA | MHOM/DZ/1996/LIPA482 | Algeria | Médéa | MON-1 | VL | 2 | na |  |
| Alg55 |  | IPA | MCAN/DZ/2001/LIPA1246 | Algeria | Alger | MON-1 | CanL | 1 | na |  |
| Alg31 |  | IPA | MHOM/DZ/2002/LIPA1313 | Algeria | Lakhdaria | MON-1 | VL | 2 | na |  |
| Alg23 |  | IPA | MCAN/DZ/2000/LIPA1139 | Algeria | Alger | MON-1 | CanL | 2 | na |  |
| INF-01 |  | CNRLM | MHOM/TN/1980/IPT1^T^ | Tunisia | nd | MON-1 | VL | 2 | na |  |
| Tun1 |  | LPMM | MHOM/TN/2001/Tus167 | Tunisia | Monastir | MON-1 | VL | 2 | na |  |
| Tun10 |  | LPMM | MHOM/TN/2002/27M | Tunisia | Tunis | MON-1 | VL | 2 | na |  |
| Tun11 |  | LPMM | MHOM/TN/2002/246M | Tunisia | Tunis | MON-1 | VL | 2 | na |  |
| Tun12 |  | LPMM | MHOM/TN/2002/20S | Tunisia | Beja | MON-1 | VL | 2 | na |  |
| Tun13 |  | LPMM | MHOM/TN/2002/22MO | Tunisia | Seliana | MON-1 | VL | 2 | na |  |
| Tun14 |  | LPMM | MHOM/TN/2002/21S | Tunisia | Béja | MON-1 | VL | 2 | na |  |
| Tun16 |  | LPMM | MHOM/TN/2002/Tus221 | Tunisia | Monastir | MON-1 | VL | 2 | na |  |
| Tun17 |  | LPMM | MHOM/TN/2002/Tum222 | Tunisia | Monastir | MON-1 | CVL | 2 | na |  |
| Tun18 |  | LPMM | MCAN/TN/2002/LCnJ20S | Tunisia | Tunis | MON-1 | CVL | 2 | na |  |
| Tun19 |  | LPMM | MCAN/TN/2002/GGCH1/02 | Tunisia | Monastir | MON-1 | CVL | 2 | na |  |
| Tun20 |  | LPMM | MCAN/TN/2002/FCH2/02 | Tunisia | Monastir | MON-1 | CVL | 2 | na |  |
| Tun21 |  | LPMM | MCAN/TN/2002/LCnJ20G | Tunisia | Tunis | MON-1 | CL | 2 | na |  |
| Tun22 |  | LPMM | MHOM/TN/2004/LC78 | Tunisia | Tunis | MON-1 | VL | 1 | na |  |
| Tun2 |  | LPMM | MHOM/TN/2002/Tus227 | Tunisia | Monastir | MON-24 | VL | 3 | na |  |
| Tun4 |  | LPMM | MHOM/TN/2005/PLV11 | Tunisia | Kairouan | MON-24 | VL | 3 | na |  |
| Tun6 |  | LPMM | MHOM/TN/2005/PLV15 | Tunisia | Kairouan | MON-24 | VL | 3 | na |  |
| Tun7 |  | LPMM | MHOM/TN/2005/PLV28 | Tunisia | Kairouan | MON-24 | CL | 3 | na |  |
| Tun23 |  | LPMM | MHOM/TN/2002/LC148 | Tunisia | Bizerte | MON-24 | CL | 3 | na |  |
| Tun24 |  | LPMM | MHOM/TN/2004/LC64 | Tunisia | Tunis | MON-24 | CL | 3 | na |  |
| Tun25 |  | LPMM | MHOM/TN/2002/LC95 | Tunisia | Béja | MON-24 | CL | 3 | na |  |
| Tun26 |  | LPMM | MHOM/TN/2002/SFC89 | Tunisia | Sfax | MON-24 | CL | 3 | na |  |
| Tun27 |  | LPMM | MHOM/TN/2005/SFC51 | Tunisia | Sfax | MON-24 | CL | 3 | na |  |
| Tun28 |  | LPMM | MHOM/TN/2004/TLC3 | Tunisia | Siliana | MON-24 | CL | 3 | na |  |
| INF-138 |  | JRCL | MCAN/UZ/2007/LRC-L1307 | Uzbekistan | Namangan | nd | CanL | 2 | na |  |
| INF-139 |  | JRCL | MCAN/UZ/2007/LRC-L1310 | Uzbekistan | Namangan | nd | CanL | 2 | na |  |
| INF-140 |  | JRCL | MCAN/UZ/2007/LRC-L1308 | Uzbekistan | Namangan | nd | CanL | 2 | na |  |
| INF-141 |  | JRCL | MCAN/UZ/2007/LRC-L1309 | Uzbekistan | Namangan | nd | CanL | 2 | na |  |
| INF-142 |  | JRCL | MCAN/UZ/2007/LRC-L1313 | Uzbekistan | Namangan | nd | CanL | 2 | na |  |
| INF-143 |  | JRCL | MCAN/UZ/2007/LRC-L1311 | Uzbekistan | Namangan | nd | CanL | 2 | na |  |
| INF-144 |  | JRCL | MCAN/UZ/2007/LRC-L1312 | Uzbekistan | Namangan | nd | CanL | 2 | na |  |
| INF-145 |  | JRCL | MCAN/UZ/2007/LRC-L1315 | Uzbekistan | Namangan | nd | CanL | 2 | na |  |
| UZ1-SLD |  | JRCL | MHOM/UZ/2007/MUA | Uzbekistan | Namangan | nd | VL | 2 | na |  |
| UZ2-SLD |  | JRCL | MHOM/UZ/2007/KOM | Uzbekistan | Namangan | nd | VL | 2 | na |  |
| UZ3-SLD |  | JRCL | MHOM/UZ/2007/ERD | Uzbekistan | Namangan | nd | VL | 2 | na |  |
| UZ4-SLD |  | JRCL | MHOM/UZ/2007/OBA | Uzbekistan | Namangan | nd | VL | 2 | na |  |
| UZ5-SLD |  | JRCL | MHOM/UZ/2007/KU | Uzbekistan | Namangan | nd | VL | 2 | na |  |
| UZ6-SLD |  | JRCL | MHOM/UZ/2007/KOM2 | Uzbekistan | Namangan | nd | VL | 2 | na |  |
| INF-05 |  | KIT | MHOM/CN/1978/D2 | China | nd | LON49 | VL | 2 | na |  |
| INF-02 |  | KIT | MHOM/CN/1954/Peking | China | nd | nd | VL | 2 | na |  |
| INF-67 |  | LSHTM | MHOM/CN/1980/StrainA | China | nd | MON-34 | nd | 2 | na |  |
| DON-01 |  | KIT | MHOM/IN/1980/DD8^T^ | India | nd | MON-2 | VL | na | na |  |
| DON-39 | LG 9 | CNRLM | MHOM/IN/0000/DEVI | India | Bihar | MON-2 | VL | na | na |  |
| DON-40 | LG 10 | CNRLM | MHOM/IN/1996/THAK35 | India | Bihar | MON-2 | nd | na | na |  |
| DON-45 |  | BHU | MHOM/IN/2001/BHU20140 | India | Bihar | nd | VL | na | na |  |
| DON-56 |  | BHU | MHOM/IN/2002/BHU6 | India | Bihar | nd | VL | na | na |  |
| DON-02 |  | KIT | MHOM/KE/1983/NLB 189 | Kenya | nd | MON-37 | PKDL | na | na |  |
| DON-03 |  | KIT | MHOM/KE/1984/NLB 218 | Kenya | nd | nd | PKDL | na | na |  |
| DON-04 |  | KIT | MHOM/KE/1985/NLB 323 | Kenya | nd | MON-37 | VL | na | na |  |
| DON-66 |  | JRCL | MHOM/KE/1955/LRC-L53 | Kenya | nd | nd | nd | na | na |  |
| DON-67 |  | JRCL | MHOM/KE/0000/LRC-L445 | Kenya | nd | nd | nd | na | na |  |
| DON-24 |  | JRCL | MHOM/ET/1967/HU3 | Ethiopia | nd | MON-18 | VL | na | na |  |
| DON-07 |  | KIT | MHOM/SD/1962/LRC-L61 | Sudan | nd | MON-82 | nd | na | na |  |
| DON-08 |  | KIT | MHOM/SD/1968/1 S | Sudan | nd | nd | VL | na | na |  |
| DON-12 |  | KIT | MHOM/SD/1992/51-band | Sudan | Gedaref | MON-30 | VL | na | na |  |
| DON-14 |  | KIT | MHOM/SD/1993/AEB | Sudan | Gedaref | MON-82 | VL | na | na |  |
| DON-13 |  | KIT | MHOM/SD/1993/GE | Sudan | Gedaref | MON-82 | VL | na | na |  |
| DON-46 | LG 24 | CNRLM | MHOM/SD/1997/LEM3429 | Sudan | Gedaref | MON-257 | VL | na | na |  |
| DON-49 | LG 17 | CNRLM | MCAN/SD/0000/LEM3946 | Sudan | Gedaref | MON-274 | VL | na | na |  |
| DON-47 | LG 25 | CNRLM | MHOM/SD/1997/LEM3463 | Sudan | Gedaref | MON-258 | VL | na | na |  |
| DON-38 | LG 13 | CNRLM | MHOM/ET/0000/HUSSEN | Ethiopia | nd | MON-31 | VL | na | na |  |
