# Supplementary material for: Comparative Microsatellite Typing of New World Leishmania infantum Reveals Low Heterogeneity among Populations and Its Recent Old World Origin
Source: PLoS Negl Trop Dis. 2011 Jun 7;5(6):e1155. doi: 10.1371/journal.pntd.0001155 (PMC3110170; doi:10.1371/journal.pntd.0001155)
Supplement: Table S2 — N, number of strains; P, proportion of polymorphic loci; MNA, mean number of alleles; Ho, observed heterozygosity; He, expected heterozygosity; F IS, inbreeding coefficient; FR – France; IT – Italy. (DOC) [file pntd.0001155.s005.doc]

**Table S2: Characterization of the main Old World *L. infantum* populations found by STRUCTURE analysis*.***

| Population | zymodeme | Origin | N | P | MNA | *H*_o_ | *H*_e_ | *F*_IS_ |
| --- | --- | --- | --- | --- | --- | --- | --- | --- |
| Pop1-INF_OW_ | MON-1 | Spain, Portugal, France, Italy | 135 | 1.000 | 5.7 | 0.033 | 0.284 | 0.883 |
| Pop2-INF_OW_ | MON-1 | Algeria, Tunisia, Greece, Turkey, China,  Uzbekistan, Israel, Palestine, few FR+IT | 121 | 1.000 | 5.4 | 0.071 | 0.420 | 0.832 |
| Pop3-INF_OW_ | non-MON-1 | Spain, Portugal, France, Italy, Malta  Algeria, Tunisia | 52 | 1.000 | 8.7 | 0.304 | 0.775 | 0.610 |
| Combined  pop1+2-INF_OW_ | MON-1 | Spain, Portugal, France, Italy, Algeria, Tunisia, Greece, Turkey, China,  Uzbekistan, Israel, Palestine | 256 | 1.000 | 7.2 | 0.051 | 0.426 | 0.881 |
