# Supplementary material for: Comparative Microsatellite Typing of New World Leishmania infantum Reveals Low Heterogeneity among Populations and Its Recent Old World Origin
Source: PLoS Negl Trop Dis. 2011 Jun 7;5(6):e1155. doi: 10.1371/journal.pntd.0001155 (PMC3110170; doi:10.1371/journal.pntd.0001155)
Supplement: Methods S1 — Additional information about the methods of MLMT data analyses. (DOC) [file pntd.0001155.s006.doc]

**Methods S1:**

**Additional information about the methods of MLMT data analyses**

Distance based methods and models based on Bayesian statistics have been applied in the analysis of the MLMT profiles.

Microsatellite based genetic distances were calculated using the Chord distance measure [1]:

*X_u_* and *Y_u_* are the frequencies of the uth allele in populations x and y, respectively. The Chord distance follows the Infinite Allele Model IAM where every new mutation is assumed to lead to a new distinguishable allele. IAM’s were the standard approaches for most allozyme analyses [2] where it was difficult or impossible to predict the state of a mutation from knowing the state of its ancestors. The alternative model of microsatellite evolution is the Stepwise Mutation Model (SMM), where alleles can only mutate by the gain or loss of one repeat unit. It has been shown that 2 bp repeat microsatellite loci (CA repeats were used in our study) are more similar to the expectations of the IAM [3] even though this model does not account for homoplasy.

The Bayesian method implemented in STRUCTURE [4] identifies genetically distinct populations, based on allele frequencies, by estimating, for each individual studied the fraction of the genotype belonging solely to it. Individuals can be assigned to multiple clusters with the membership coefficients of all those clusters summing up to one. This clustering method proved superior to distance-based approaches for processing data sets of low variability. However, panmixia is one of the essential assumptions in the STRUCTURE algorithm. Nevertheless, even if used with organisms not in Hardy-Weinberg equilibrium, STRUCTURE results have so far always corroborated those obtained by genetic distance, have accurately inferred individual ancestries, have been appropriate for characterization of population structure and have provided information on population relationships and history [5,6,7].

*F_ST_*, is a measure of genetic differentiation between populations, and *F_IS_*, is a measure of the level of inbreeding within a population. These parameters provide valuable information about population differentiation and the mode of reproduction [8].

*F_ST_* has a theoretical minimum of 0, meaning no genetic structure or differentiation, and a theoretical maximum of 1, meaning complete differentiation. The observed maximum is usually much less than 1. The following guidelines have been suggested for the interpretation of *F_ST_* [9]:

*F_ST_* = 0 - 0.05, little, but by no means negligible, genetic differentiation

*F_ST_* = 0.05 - 0.15, moderate genetic differentiation

*F_ST_* = 0.15 -0.25, great genetic differentiation

*F_ST_* = > 0.25, very great genetic differentiation

The inbreeding coefficient *F_IS_* measures the inbreeding of individuals that is due to the local non-random union of gametes in each subpopulation and is most informative for testing the deviation from random mating in subpopulations [10,11]. If *F*_IS_ = 0 the expectations of random mating in Hardy-Weinberg are met, whereas *F*_IS_ = 1 means complete inbreeding and *F*_IS_ = -1 complete outbreeding. Clonal diploids are expected to accumulate heterozygosity over time at every locus and should therefore exhibit negative *F_is_* values. Heterozygote deficiency (positive *F_is_* values) seen in many MLMT studies of *Leishmania* can be due to the presence of different factors, such as population subdivision (Wahlund effect) or a high rate of gene conversion. The high *F_is_* values found in MLMT analyses of Bolivian and Peruvian *L. braziliensis* [12] and in Ethiopian *L. donovani* [13] could be only partly explained by population subdivision.

1. Cavalli-Sforza LL, Edwards AW (1967) Phylogenetic analysis. Models and estimation procedures. Am J Hum Genet 19: 233-257.

2. Nei M (1984) Molecular Evolutionary Genetics. New York: Columbia University Press.

3. Shriver MD, Jin L, Chakraborty R, Boerwinkle E (1993) VNTR allele frequency distributions under the stepwise mutation model: a computer simulation approach. Genetics 134: 983-993.

4. Pritchard JK, Stephens M, Donnelly P (2000) Inference of population structure using multilocus genotype data. Genetics 155: 945-959.

5. Al-Jawabreh A, Diezmann S, Mueller M, Wirth T, Schnur LF, et al. (2008) Identification of geographically distributed sub-populations of *Leishmania* (*Leishmania*) *major* by microsatellite analysis. BMC Evol Biol 8: 183.

6. Wirth T, Hildebrand F, Allix-Beguec C, Wolbeling F, Kubica T, et al. (2008) Origin, spread and demography of the *Mycobacterium tuberculosis* complex. PLoS Pathog 4: e1000160.

7. Schwenkenbecher JM, Wirth T, Schnur LF, Jaffe CL, Schallig H, et al. (2006) Microsatellite analysis reveals genetic structure of *Leishmania tropica*. Int J Parasitol 36: 237-246.

8. De Meeus T, Lehmann L, Balloux F (2006) Molecular epidemiology of clonal diploids: a quick overview and a short DIY (do it yourself) notice. Infect Genet Evol 6: 163-170.

9. Wright S (1978) Evolution and the Genetics of Populations. Variability within and among Natural Populations. Chikago: University of Chikago Press.

10. Halkett F, Simon JC, Balloux F (2005) Tackling the population genetics of clonal and partially clonal organisms. Trends Ecol Evol 20: 194-201.

11. de Meeus T, McCoy KD, Prugnolle F, Chevillon C, Durand P, et al. (2007) Population genetics and molecular epidemiology or how to "debusquer la bete". Infect Genet Evol 7: 308-332.

12. Rougeron V, De Meeus T, Hide M, Waleckx E, Bermudez H, et al. (2009) Extreme inbreeding in *Leishmania braziliensis*. Proc Natl Acad Sci U S A 106: 10224-10229.

13. Gelanew T, Kuhls K, Hurissa Z, Weldegebreal T, Hailu W, et al. (2010) Inference of population structure of *Leishmania donovani* strains isolated from different Ethiopian visceral leishmaniasis endemic areas. PLoS Negl Trop Dis 4: e889.
